# Agrobacterium-derived cytokinin influences plastid morphology and starch accumulation in Nicotiana benthamiana during transient assays

**DOI:** 10.1186/1471-2229-14-127

**Published:** 2014-05-09

**Authors:** Jessica L Erickson, Jörg Ziegler, David Guevara, Steffen Abel, Ralf B Klösgen, Jaideep Mathur, Steven J Rothstein, Martin H Schattat

**Affiliations:** 1 Abteilung Pflanzen Physiologie, Institut für Biologie-Pflanzenphysiologie, Martin-Luther-Universität Halle-Wittenberg, Weinbergweg 10, Halle/Saale 06120, Germany; 2 Abteilung Molekulare Signalverarbeitung, Leibniz-Institut für Pflanzenbiochemie, Weinberg 3, Halle/Saale 06120, Germany; 3 Present Address: Pioneer Hi-Bred, 12111 Mississauga Rd, Georgetown, ON L7G 4S7, Canada; 4 Department of Molecular and Cellular Biology, University of Guelph, Guelph, ON N1G 2 W1, Canada

**Keywords:** *Agrobacterium tumefaciens*, *Nicotiana benthamiana*, Transient assays, GV3101(pMP90), LBA4404, Plastid, Stromules, Bacteria-derived, Cytokinin, *Trans*-zeatin synthase

## Abstract

**Background:**

*Agrobacterium tumefaciens*-based transient assays have become a common tool for answering questions related to protein localization and gene expression in a cellular context. The use of these assays assumes that the transiently transformed cells are observed under relatively authentic physiological conditions and maintain ‘normal’ sub-cellular behaviour. Although this premise is widely accepted, the question of whether cellular organization and organelle morphology is altered in *Agrobacterium*-infiltrated cells has not been examined in detail. The first indications of an altered sub-cellular environment came from our observation that a common laboratory strain, GV3101(pMP90), caused a drastic increase in stromule frequency. Stromules, or ‘stroma-filled-tubules’ emanate from the surface of plastids and are sensitive to a variety of biotic and abiotic stresses. Starting from this observation, the goal of our experiments was to further characterize the changes to the cell resulting from short-term bacterial infestation, and to identify the factor responsible for eliciting these changes.

**Results:**

Using a protocol typical of transient assays we evaluated the impact of GV3101(pMP90) infiltration on chloroplast behaviour and morphology in *Nicotiana benthamiana*. Our experiments confirmed that GV3101(pMP90) consistently induces stromules and alters plastid position relative to the nucleus. These effects were found to be the result of strain-dependant secretion of cytokinin and its accumulation in the plant tissue. Bacterial production of the hormone was found to be dependant on the presence of a *trans*-zeatin synthase gene (*tzs*) located on the Ti plasmid of GV3101(pMP90). Bacteria-derived cytokinins were also correlated with changes to both soluble sugar level and starch accumulation.

**Conclusion:**

Although we have chosen to focus on how transient *Agrobacterium* infestation alters plastid based parameters, these changes to the morphology and position of a single organelle, combined with the measured increases in sugar and starch content, suggest global changes to cell physiology. This indicates that cells visualized during transient assays may not be as ‘normal’ as was previously assumed. Our results suggest that the impact of the bacteria can be minimized by choosing *Agrobacterium* strains devoid of the *tzs* gene, but that the alterations to sub-cellular organization and cell carbohydrate status cannot be completely avoided using this strategy.

## Background

The soil-borne bacterium *Agrobacterium tumefaciens* is the cause of crown gall disease in various plant species. The Ti-plasmid of virulent *A. tumefaciens* strains is essential for tumor induction during bacterial infection. A distinct part of this plasmid (the T-DNA) is excised and transferred to the plant cell via a type IV secretion apparatus, then transported to the nucleus where it is finally inserted into the plant genome. The transferred wild type T-DNA encodes genes that force the transformed plant cell to synthesize the plant hormones auxin and cytokinin as well as amino acid–sugar conjugates (opines). The resulting increase in auxin and cytokinin levels in the plant tissue induces cell proliferation resulting in tumor formation. The opines, which are produced only by transformed cells, are utilized by *A. tumefaciens* as a carbon and nitrogen source. The type of opine(s) produced is used to classify the infectious *A. tumefaciens* strains as octopine, nopaline or agropine-type strains (reviewed in [1-4]).

The removal of the T-DNA region of wild type Ti-plasmids yields bacterial strains that are no longer capable of stimulating tumor formation or opine production in plant cells. In place of the wild type, tumor inducing, T-DNA, modified T-DNAs located on binary T-DNA vectors have been designed and utilized to efficiently mediate the transfer of genes to plant cells (reviewed in [1,5]). The ability to transfer genes of interest to plant genomes without inducing tumors made such ‘disarmed’ *A. tumefaciens* strains invaluable to plant gene technology (reviewed in [2,4,6]).

Today *A. tumefaciens* is routinely used to generate transgenic plant lines for biotechnology or research purposes. However, the process of establishing transgenic lines is time consuming, and for several applications, not mandatory. The use of *A. tumefaciens* in transient assays provides a time saving alternative to the generation of stable transgenic plants. In most of these assays, ‘disarmed’ *A. tumefaciens* suspensions containing the construct of interest are infiltrated into leaf tissue. Cells exposed to the bacteria are subsequently transformed with the sequence of interest and can be assayed for expression a few days after infiltration. This method of plant cell transformation is often preferable to particle bombardment as it introduces fewer copies of the sequence, with a lower frequency of rearrangement (reviewed in [7]). Based on their high transformation efficiency, leaves of *Nicotiana tabacum* and *Nicotiana benthamiana* are commonly used for *A. tumefaciens* mediated transient expression.

Although infiltration of *N. tabacum* and *N. benthamiana* with ‘disarmed’ *A. tumefaciens* causes seemingly minor macroscopic effects [8], there is a growing body of evidence suggesting that even ‘disarmed’ strains are recognized by these *Nicotiana* species as a pathogen, and that pathogen related responses can interfere with transient gene expression assays [8,9]. Although the mechanism of these interactions is not well understood, it clearly indicates that the use of *A. tumefaciens* in a transient system has limitations. The use of such assays is based on the assumption that plant cells expressing the gene(s) of interest are maintaining authentic sub-cellular behaviour. However, aside from the mentioned effect of *A. tumefaciens* on pathogen related responses, the impact of the bacteria on other aspects of cell biology, such as protein localisation, organelle movement and organelle morphology, has not been studied or reported in detail. During standard transient assays using the ‘disarmed’ laboratory strain GV3101(pMP90) it became evident to us that *A. tumefaciens* can indeed have a pronounced effect on organelle morphology in *N. benthamiana*. Specifically, we observed that infiltration with this bacterial strain lead to the increased formation of stroma-filled-tubules (stromules) emanating from the surface of plastids and seemed to alter plastid position relative to the nucleus [10].

Stromules are approximately 0.1 to 0.8 μm in diameter (reviewed in [11]) and can range from only a few μm to 45 μm in length [12]. They are a common morphological feature of all plastid types, and have been observed in both vascular and non-vascular plants (monocotyledons, dicotyledons, moss and green algae) (reviewed in [13]), suggesting that these structures have been conserved during *Viridiplantae* evolution. It is known that stromules form at certain developmental stages, and the frequency of these protrusions is elevated when plants are exposed to a variety of stresses (biotic as well as abiotic) (reviewed in [14]). Although this suggests that stromules support plant cells in coping with unfavorable conditions, the specific subcellular function remains speculative.

Based on the sensitivity of stromule formation to stress, we interpreted our initial observation that GV3101(pMP90) induces stromules as a first indication of potential sub-cellular changes induced by short-term infestation of *A. tumefaciens* during transient assays. Our goal was to identify the specific elicitor responsible for the observed changes, thus better understanding how the ‘disarmed’ strain alters the sub-cellular environment of *N. benthamiana*, and simultaneously gain insight into the phenomenon of stromule formation.

Our results have confirmed that GV3101(pMP90) reliably induces stromules, and additionally, we have observed changes in plastid positioning relative to the nucleus following bacterial infiltration. These changes induced by GV3101(pMP90) are strain specific, and were found to be dependant on the presence of a Ti-plasmid specific *trans-zeatin synthase* gene (*tzs*). Further, we demonstrate that the production of cytokinins by the bacteria during transient assays is sufficient to alter cell physiological status, increasing both soluble sugar level and starch accumulation and that this can partially be avoided by utilizing alternative ‘disarmed’ strains.

## Results

To study the effect of *A. tumefaciens* infiltration on plastid behavior and morphology we utilized transgenic *N. benthamiana* lines constitutively expressing the chimeric protein FNR-EGFP, which highlights the plastid stroma and stromules [15,16]. Using these stable transgenic lines the effect of *A. tumefaciens* infiltration can be easily estimated by comparison of infiltrated and non-infiltrated tissue using fluorescence microscopy. To simplify the description of the results we have assigned acronyms to all bacterial strains utilized and listed them in Table 1, along with their antibiotic resistance.

**Table 1:** **Antibiotic resistance of *Agrobacterium tumefaciens *** **strains**

***A.tumefaciens *** **strain**				
**Full strain name**	**Acronym**	**Genome**	**Ti Plasmid**	**T-DNA vector**
GV3101(pMP90)	GV	Rif	Gent	-
GV3101(pMP90)/pCP60-35S-DsRed2	GVR	Rif	Gent	Kan
LBA4404	LBA	Rif	Strep	-
LBA4404/pCP60-35S-DsRed2	LBR	Rif	Strep	Kan
GV3101/pCP60-35S-DsRed2 (cured)	GVC	Rif	-	Kan
LBA/pLSU-*ptzs*-*tzs*	LtZ	Rif	Strep	Kan

Disarmed *A. tumefaciens* strains (column 1), acronyms used (column 2) and location of the genes conferring resistance to the various antibiotics, this includes the resistance located in the bacterial genome (column 3), the Ti plasmid (column 4) and the T-DNA vector (column 5). Antibiotics are abbreviated as follows: Rif = rifampicin, Gent = gentamicin, Strep = Streptomycin, Kan = kanamycin, and the absence of the T-DNA vector or Ti plasmid is indicated by a dash (-).

### Infiltration with GVR induces stromule formation as well as plastid repositioning

**Figure 1:**
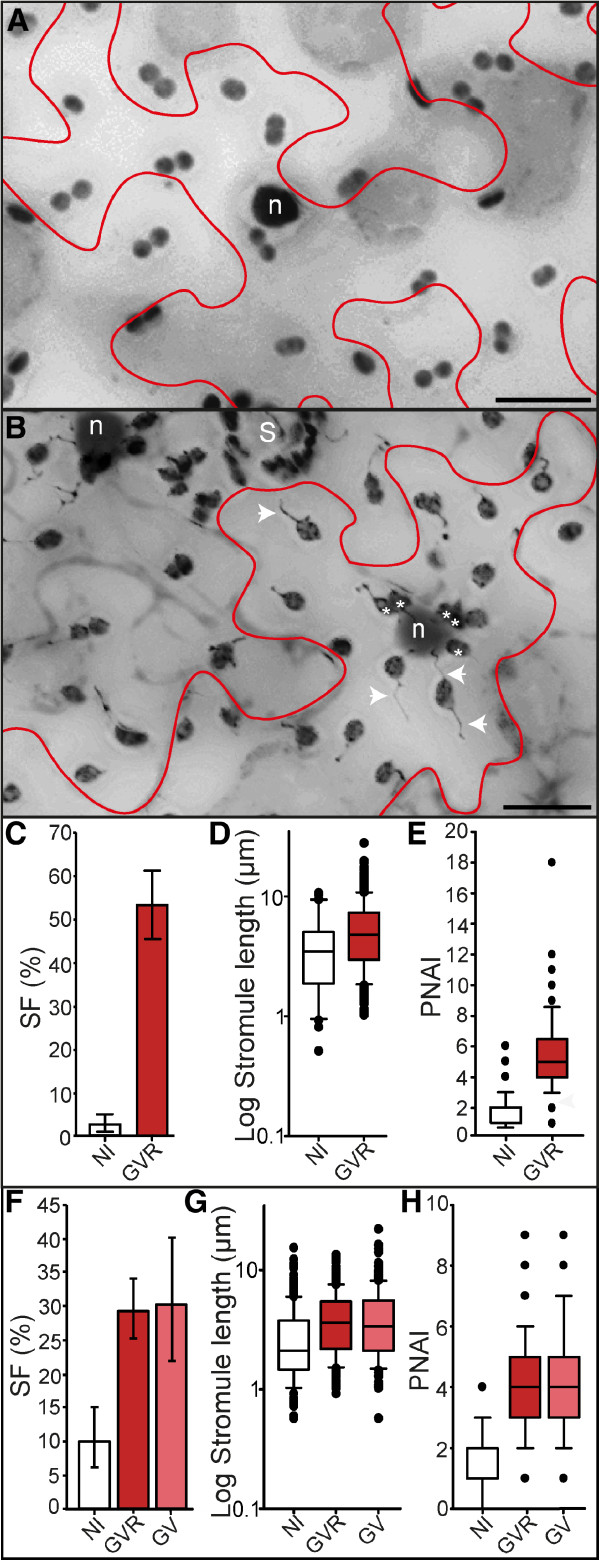
**Impact of infiltration with *A.tumefaciens *strains GVR and GV on stromule induction and plastid position.** ‘Stacked’ fluorescence images of *N. benthamiana* lower epidermis with FNR-EGFP labeled plastids (single cells outlined in red). Nuclei of non-infiltrated cells were labeled via DAPI staining, while the nuclei of GVR-infiltrated cells were labeled via nucleoplasmic DsRed2. Epidermal plastids are dark grey, nuclei labeled ‘n’, location of stomata labeled ‘S’ (plastids surrounding are within guard cells). Plastids in close proximity to the nucleus are indicated by asterisks ‘*’. Images were converted to gray scale and inverted for easier viewing of stromules. Images were taken 3 days post-infiltration. Scale bars=20 μm **(A, B)**. **A** Non-infiltrated tissue. **B** GVR-infiltrated tissue. Examples of stromules indicated with white arrows. **C** Bar graph illustrating average stromule frequency (SF) in non-infiltrated (NI) and GVR-infiltrated (GVR) tissues. Rank sum (NI-GVR): U=0, p<0.001. Sample sizes: n(NI,GVR)=9. **D** Box plot illustrating the distribution and median stromule lengths in non-infiltrated (NI) and GVR-infiltrated (GVR) tissues. Rank sum (NI-GVR): U=3002, p=0.022. Sample sizes: n(NI)=30, n(GVR)=269. **E** Box plot illustrating median plastid nuclear association index (PNAI) in non-infiltrated (NI) and GVR-infiltrated (GVR) tissues. Rank sum (NI-GVR): U=593, p<0.001. Sample sizes: n(NI)=100, n(GVR)=73. **F** Bar graph illustrating average stromule frequency (SF) in non-infiltrated (NI), GVR-infiltrated (GVR) and GV-infiltrated (GV) tissues. Rank sum (NI-GVR): U=1, p<0.001; (NI-GV): U=4, p=0.001; (GV-GVR): U=34, p=0.596. Sample sizes: n(NI,GV,GVR)=9. **G** Box plot of median stromule lengths in non-infiltrated (NI), GVR-infiltrated (GVR) and GV-infiltrated (GV) tissues. Rank sum (NI-GVR): U=56132, p<0.001); (NI-GV): U=28578.5, p<0.001; (GV-GVR): U=68971.5, p=0.652). Sample sizes: n(NI)=325, n(GVR)=535, and n(GV)=263. **H** Box plot illustrating the median plastid nuclear association index (PNAI) in non-infiltrated (NI), GVR-infiltrated (GVR) and GV-infiltrated (GV) tissues. Rank sum (NI-GVR): U=7356.5, p<0.001; (NI-GV): U=6266, p<0.001; (GV-GVR): U=12097.5, p=0.082. Sample sizes: n(NI)=259, n(GVR)=157, and n(GV)=173. **C, F** Raw data was arcsine transformed and bars represent back-transformed means. Error bars represent back-transformed 95% confidence intervals. **D, E, G, H** Boxes contain 50% of data, the median is represented by black line, and error bars represent 90% confidence intervals.

A ‘disarmed’ *A. tumefaciens* strain, **GV**3101(pMP90) (abbreviated - **GV**) [17] was employed in this study due to its wide-spread use for transient gene expression in *N. benthamiana*. In order to monitor transformation activity of *A. tumefaciens,* GV was transformed with the binary vector pCP60-35S-DsRed2 to yield GV3101(pMP90)/pCP60-35S-DsRed2 (abbreviated – **GVR**). This vector facilitates the expression of untagged DsRed2 [10]. Three days after infiltration, DsRed2 was detectable in infiltrated areas as bright fluorescence signals in the cytoplasm and nucleoplasm, indicating successful transfer and expression of the reporter gene from *A. tumefaciens* to the plant (Additional file 1: Figure S1C). In contrast, cells of non-infiltrated areas did not show any fluorescence signal using the same or even more sensitive microscope settings (Additional file 1: Figure SD and S1F respectively).In the leaves of FNR-EGFP transgenic plants the plastids are clearly highlighted by the EGFP fluorescence. Following infiltration with GVR there were drastic alterations to plastid morphology and plastid position when compared to untreated tissue. The most obvious difference following GVR treatment was the large number of stromules compared to the control (Figure 1A and 1B). The average stromule frequency (SF) in GVR-infiltrated leaf areas was approximately 53%, which was significantly higher than the 3% observed in non-infiltrated tissues (Figure 1C).

Stromule morphology also appeared altered in cells infiltrated with GVR. In non-infiltrated tissues there was an abundance of short stromules, mostly in the range from 3 to 5 μm, only in rare cases exceeding 10 μm. In contrast to this, in cells which were exposed to GVR, 50% of stromules demonstrated lengths of 5 μm or higher, with a maximum length of approximately 27 μm (Figure 1D). In addition to exhibiting substantially longer stromules, there was also frequent observation of crooked and branched stromules (Figure 1B). These branches originate from triangular dilations along the ‘main stromule tubule’ and were described in earlier publications [16,18].

It was also observed that, in many GVR-infiltrated cells, a subpopulation of plastids clustered around the nucleus (Figure 1B) in contrast to the untreated tissue, in which plastids are largely observed as part of evenly distributed pairs (Figure 1A). For quantification of this parameter we counted the number of plastids associated with individual nuclei, which we have named ‘**P**lastid-**N**uclear **A**ssociation **I**ndex’ (PNAI). Nuclei were labeled via DsRed2 accumulation in the nucleoplasm of GVR-infiltrated tissue, whereas in untreated tissue nuclei were detected after incubating the leaf disks in DAPI for 10 minutes. The box plot in Figure 1E clearly shows that after GVR treatment, the number of plastids in close proximity to the nucleus increases significantly, changing from a PNAI median of 2 (minimum of 1; maximum of 6) in untreated areas, to a PNAI median of 5 in infiltrated tissues (minimum 1; maximum of 18).

Our initial experiments confirmed the previous observation that the chosen strain, GVR, induces drastic changes in stromule frequency. In control experiments performed previously [10], the infiltration itself, and the agrobacterium infiltration medium (AIM) used for solubilizing the bacteria, were ruled out as the stromule inducing stimuli, providing evidence that the inducing factor most likely originates from the bacteria. The goal of the second set of experiments was to identify the bacterial factor responsible for these ‘stromule inducing’ changes to the sub-cellular environment.

### The expression of the reporter protein has no influence on the observed changes that follow GVR infiltration

The over-expression of proteins in stable as well as transient systems has the potential to interfere with normal cell metabolism as well as organelle behaviour [19-21]. In order to test if overexpression of the reporter protein was responsible for the observed effects, infiltration experiments were repeated utilizing GV lacking the pCP60-DsRed2 T-DNA vector. As a positive control we also infiltrated with GVR into the same leaves. GV and GVR-infiltrated tissues showed comparable levels of stromule induction, with both treatments yielding significantly higher SF values compared to the non-treated areas (Figure 1F). There was also no significant difference in stromule length (Figure 1G), or PNAI values (Figure 1H). Like GVR-infiltrated tissues, GV infiltration resulted in a higher tendency of plastids to associate with the nucleus (PNAI median of 4, minimum 1; maximum 9) when compared to untreated cells (Figure 1H). These results demonstrate that the transient expression of DsRed2 is not the cause for the GVR induced changes, but confirms that the source of stromule induction is intrinsic to the bacteria itself. GV is a disarmed derivative of the nopaline wild-type strain C58, which is the parental isolate for many disarmed laboratory strains (reviewed in [5]). We repeated the experiments using a different *A. tumefaciens* lab strain, originating from a different wild type isolate.

### Infiltration of LBA and LBR does not induce plastid morphology and position changes to the same extent as GV and GVR

**Figure 2:**
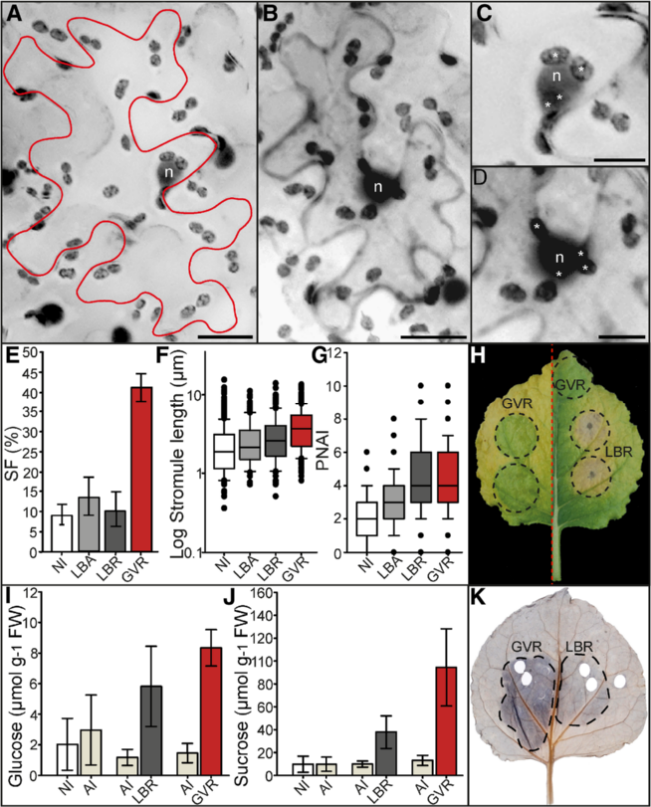
**Infiltration with LBA and LBR induces mild alterations to plastid morphology and position relative to GVR infiltrated cells.** ‘Stacked’ fluorescence images of *Nicotiana benthemiana* lower epidermis with FNR-EGFP labeled plastids. In non-infiltrated and LBA-infiltrated treatments nuclei were stained via DAPI, while nuclei of LBR and GVR treatments were labeled via nucleoplasmic DsRed2. Epidermal plastids are dark grey, nuclei labeled ‘n’. Images were converted to gray scale and inverted for easier viewing of stromules. Images were taken 3 days post-infiltration. Scale=20 μm **(A, B)**, or 10 μm **(C, D)**. **A** Infiltrated with LBA. Cell boundary marked in red. **B** Infiltrated with LBR. Cell boundary marked by cytosolic DsRed2. **C, D** Enlargements of nuclei in **A** and **B** respectively, showing plastids in close proximity to the nucleus (indicated by asterisks ‘*’). **E** Bar graph of mean stromule frequency (SF) in non-infiltrated (NI), LBA-infiltrated (LBA), LBR-infiltrated (LBR), and GVR-infiltrated (GVR) tissues. Rank sum (NI-LBA): U=48, p=0.131; (NI-LBR): U=73, p=0.872; (LBA-LBR): U=28, p=0.289); (GVR-NI, LBA and LBR): U=0, p<0.001. Sample sizes: n(NI)=17, n(LBA)=9, n(LBR)=9, and n(GVR)=18. Raw data were arcsine transformed and bars represent back-transformed means. Error bars represent back-transformed 95% confidence intervals. **F** Box plot illustrating median stromule lengths in non-infiltrated (NI), LBA-infiltrated (LBA), LBR-infiltrated (LBR), and GVR-infiltrated (GVR) tissues. Rank sum (NI-LBA): U=34726, p=0.005; (NI-LBR): U=25226.5, p<0.001; (LBA-LBR): U=11983.5, p=0.053; (NI-GVR): U=95386.5, p<0.001; (LBA-GVR): U=46698, p<0.001; (LBR-GVR): U=45923, p<0.001. Sample sizes: n(NI)=448, n(LBA)=181, n(LBR)=151, and n(GVR)=805. **G** Box plot illustrating median plastid nuclear association index (PNAI) in non-infiltrated (NI), LBA-infiltrated (LBA), LBR-infiltrated (LBR), and GVR-infiltrated (GVR) treatments. Rank sum (NI-LBA): U=8772, p<0.001; (LBA-LBR): U=3380.5, p<0.001; (LBR-NI): U=2384, p<0.001; (GVR-NI): U=7738, p<0.001; (GVR-LBA): U=10906, p<0.001); (LBR-GVR): U=9450.5, p=0.359. Sample sizes: n(NI)=182, n(LBA)=147, n(LBR)=83, and n(GVR)=244. **F, G** Boxes contain 50% of data, the median is represented by black line. Error bars represent 90% confidence intervals. **H** Two senescing leaves (divided by a red dotted line) showing the maintenance of ‘green islands’ in GVR-infiltrated regions, and premature senescence of LBR-infiltrated regions. Infiltration zones outlined with black dotted lines. **I** Mean absolute glucose content of non-infiltrated (NI), AIM-infiltrated (AI), LBR-infiltrated (LBR) and GVR-infiltrated (GVR) tissues. Rank sum (NI-AI): U=14, p=0.209; (AI-LBR): U=0, p<0.001, t-test (AIM-GVR): t=13.577, p<0.001; (LBR-GVR): t=2.323, p=0.039. Sample sizes: n(NI, AI, LBR, GVR)=7. Error bars represent standard deviations. **J** Mean absolute sucrose content of non-infiltrated (NI), AIM-infiltrated (AI), LBR-infiltrated (LBR) and GVR-infiltrated (GVR) tissues. Rank sum (NI-AI): U=24, p=1, t-test (AIM-LBR): t=5.082, p<0.001, Rank sum (AIM-GVR): U=0, p<0.001), t-test (LBR-GVR): t=4.098, p=0.001). Sample sizes: n(NI, AI, LBR, GVR)=7. Error bars represent standard deviations. **K** Starch staining of a leaf infiltrated with GVR and LBR 3 days post-infiltration. Infiltration zones outlined with black dotted lines.

In order to evaluate whether the capacity for stromule induction was specific to C58 derived strains, or was more likely to be a general characteristic of *A. tumefaciens*, we transformed **LB**A4404 with pCP60-35S-Ds**R**ed2 (abbreviated - **LBR**) and repeated the experiments as outlined for GVR. **LBA**4404 (abbreviated – **LBA**) is one of the few ‘disarmed’ lab strains derived from the octopine wild type Ach5, a different wild type isolate than C58 (reviewed in [5]). Furthermore, to exclude an influence on the phenotype by over-expression of the reporter protein we also infiltrated with untransformed LBA. Again GVR was infiltrated into the same leaves to act as positive control for stromule induction.In comparison to GV and GVR, LBA and LBR-infiltrated tissue had significantly fewer stromules, and cells looked much like non-infiltrated cells (Figure 2A, 2B and 2E). However, stromules observed after LBA and LBR treatments were slightly, but significantly longer (medians of 2.11 and 2.58 μm, respectively) than in non-infiltrated tissues (median of 1.86 μm), though still shorter than after GVR treatment (median of 3.68 μm) (Figure 2F). PNAI results were very similar (Figure 2G). Clustering of plastids around the nucleus was observed more frequently in both LBA treatments when compared to untreated tissue (Figure 2C and 2D). Notably the PNAI of LBR-infiltrated tissue was not significantly different from that in the GVR treatment (Figure 2G).

SF measurements clearly show that strong induction of stromules is specifically induced by GV and GVR but not by LBA and LBR strains. However, LBA and LBR infiltrations still have an effect on the respective tissue, which is reflected in stromule length and the PNAI. Based on the chosen parameters, this indicated that the presence of LBA and LBR also changes cell physiology, but to a lesser extent than GV and GVR. The stronger effects of GV and GVR infiltration on stromule induction and plastid repositioning compared to those obtained with LBA and LBR infiltration suggest that they are due to differences in either the bacterial chromosome or the disarmed Ti-plasmid present in these strains.

### GVR-infiltrated tissues exhibit symptoms indicative of elevated cytokinin

Another phenomenon observed after infiltration concerns the senescence of infiltrated leaf tissue, which is detected as yellowing of leaf tissue approximately 6 weeks post-infiltration. GVR-infiltrated tissues were maintained as green islands (Figure 2H) in a manner reminiscent of cytokinin treatments described in the literature (reviewed in [22]). Infiltration with AIM buffer or LBR did not show the same effect. While GVR treatment delayed tissue senescence, infiltration with LBR actually induced a premature yellowing and, in some cases, even necrosis starting from the point of infiltration (Figure 2H). AIM infiltration caused neither the formation of green islands, nor premature senescence (data not shown). The ability of GVR to induce the formation of green islands suggests the possible accumulation of cytokinins in tissue infiltrated with this particular bacterial strain.

An alternative phenomenon after treatment of tobacco cell cultures with cytokinins is the pronounced and immediate accumulation of starch in plastids, as visualized by iodine staining [23,24]. The accumulation of starch was analyzed in GVR as well as LBR-infiltrated tissue by iodine staining three days post-infiltration. The result was that GVR-infiltrated areas showed a darker staining than the surrounding non-infiltrated tissue, indicating high levels of starch accumulation (Figure 2K). Microscopy of starch stained epidermal cells showed that this accumulation also took place in cells of the lower epidermis and is not restricted to mesophyll tissue (data not shown). Re-evaluation of fluorescence images taken for previous experiments revealed that starch accumulation was visible in GVR as well as in GV treatments as EGFP and chlorophyll fluorescence free areas in the plastid stroma (data not shown). LBA-infiltrated areas were, in most cases, indistinguishable from non-infiltrated leaf tissue. In rare cases infiltration with LBR showed a slightly darker staining than the surrounding tissue (Figure 2K). The pronounced accumulation of starch in GVR and GV compared to the low accumulation in LBA treatments, indicates that cytokinin accumulation is specifically induced following infiltration with GVR and GV, and that this effect is not common to all *A. tumefaciens* strains.

Cytokinins are known to be involved in the regulation of source-sink relationships. In addition to altering starch accumulation this could also be reflected by a change in soluble sugar levels [25,26]. In order to test if soluble sugar levels are influenced by *A. tumefaciens* treatment, glucose and sucrose concentration of GVR, LBR, AIM and non-infiltrated tissues was measured (Figure 2I and 2J). Infiltration with AIM does not induce any significant changes in the sugar concentrations when compared to non-infiltrated tissues (~3 μmol g^-1^ FW glucose and ~ 10 μmol g^-1^ FW sucrose after AIM treatment, compared to non-infiltrated tissues which had ~ 2 μmol g^-1^ FW glucose and ~ 10 μmol g^-1^ FW sucrose). In contrast to AIM infiltration, both LBR and GVR induced clear and significant increases in glucose as well as sucrose concentration. LBR infiltration induced a 5-fold increase in glucose concentration (5.8 μmol g^-1^ FW glucose), and a 4-fold increase in sucrose (37.7 μmol g^-1^ FW sucrose). GVR infiltration induced a greater increase in soluble sugars, with a 5.5-fold increase in glucose concentration (8.3 μmol g^-1^ FW glucose) and a 7-fold increase in sucrose concentration (94.46 μmol g^-1^ FW sucrose). As already seen for the other parameters the induced glucose and sucrose accumulation is more severe for GVR than as it is for LBR. Comparing both *A. tumefaciens* strains it becomes increasingly evident that GV and GVR strains induce more severe physiological changes than LBA and LBR.

### Cured GVR fails to induce stromule formation, plastid relocation and cytokinin related effects

**Figure 3:**
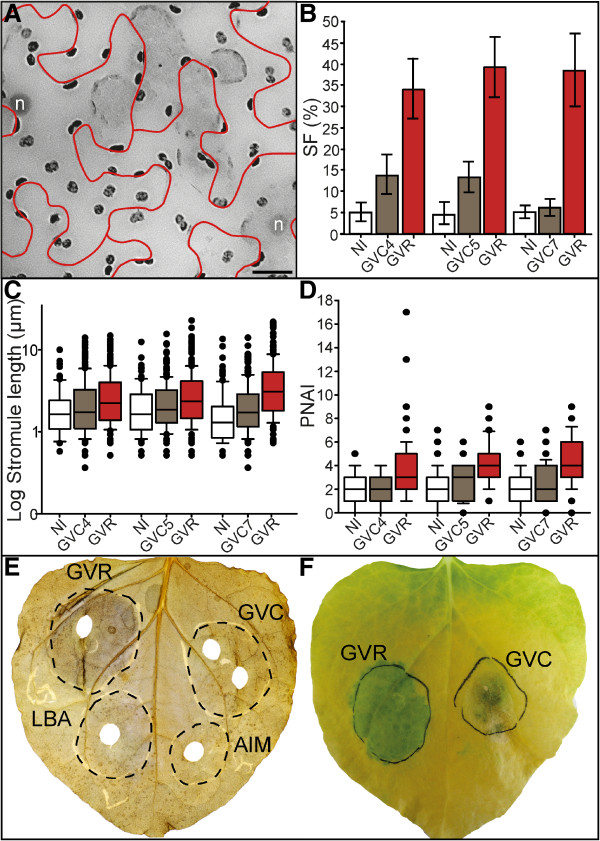
**Curing GVR of the Ti plasmid (pMP90) reduces the severity of effects seen following GVR infiltration. A** ‘Stacked’ fluorescence image of the lower epidermis of FNR-GFP transgenic *N. benthamiana* three days after infiltration with a GVC strain (lacking the Ti plasmid). Cells are outlined in red. Nuclei were labeled via DAPI (labeled ‘n’). Image was converted to gray scale and inverted to better visualize plastids. Scale=20 μm. **B, C, D** Stromule frequency (SF), stromule length, and PNAI data were collected for three different cured strains, each infiltrated into three different *N. benthamiana* plants. **B** Bar graph representing average stromule frequency (SF) in non-infiltrated (NI) tissue, and following infiltration with GVC (GVC4, 5, 7) and GVR (GVR). Rank sum (GVR-NI, GVC5, GVC7): U=0, p<0.001; (GVR-GVC4): U=1, p<0.001; (NI-GVC4): U=5, p=0.002; (NI-GVC5): U=6, p=0.002; (NI-GVC7): U=32, p=0.480). Sample sizes: n(NI, GVR paired with GVC4 and GVC5, GVC4, GVC7)=9, n(GVC5)=10, n(GVR paired with GVC7)=8. Raw data were arcsine transformed and bars represent back-transformed means. Error bars represent back-transformed 95% confidence intervals. **C** Box plot representing median stromule lengths in non-infiltrated (NI), GVC-infiltrated (GVC4, 5, and 7) and GVR-infiltrated (GVR) tissues. Rank sum (NI-GVC4): U=9837.5, p=0.051; (NI-GVC7): U=8385, p=0.178; (GVC5-NI): U=9685, p<0.001; (GVC4-GVR): U=27315.5, p=0.002; (GVC5-GVR): U=19829, p<0.001; (GVC7-GVR): U=17773.5, p=0.007; (GVR-NI paired with GVC4): U=9366.5, p<0.001; (GVR-NI paired with GVC5): U=5904, p<0.001; (GVR-NI paired with GVC7): U=9512, p<0.001. Sample sizes (consecutively): n(NI)=95, n(GVC4)=240, n(GVR)=270, n(NI)=104, n(GVC5)=245, n(GVR)=270, n(NI)=106, n(GVC7)=175 and n(GVR)=240. **D** Box plot representing median plastid nuclear association index (PNAI) in non-infiltrated (NI), GVC-infiltrated (GVC4, 5, and 7), and GVR-infiltrated (GVR) tissues. Rank sum (NI-GVC4): U=2260, p=0.357; (NI-GVC7): U=1931.5, p=0.105; (GVC5-NI): U=3344, p=0.031; (GVC4-GVR): U=1446, p<0.001; (GVC5-GVR): U=1421, p<0.001; (GVC7-GVR): U=1010.5, p<0.001; (GVR-NI paired with GVC4): U=1370, p<0.001; (GVR-NI paired with GVC5): U=1272.5, p<0.001; (GVR-NI paired with GVC7): U=608.5, p<0.001). Sample sizes (consecutively): n(NI)=74, n(GVC4)=67, n(GVR)=75, n(NI)=94, n(GVC5)=87, n(GVR)=80, n(NI)=62, n(GVC7)=74, and n(GVR)=56. **C, D C,D** Boxes contain 50% of data, the median is represented by black line. Error bars represent 90% confidence intervals. **E** Iodine stained leaf three days after infiltration with GVR, GVC4 (labeled GVC), LBA, and the buffer only control (labeled AIM). Infiltrated areas marked by a black dotted outline. **F** Infiltrated leaf at the beginning of senescence. Regions infiltrated with GVR remain green as leaf begins to senescence, forming ‘green islands’, while GVC-infiltrated tissue shows accelerated senescence.

In order to evaluate the possible differences between the GV and LBA strains that could be responsible for the observed physiological effects we reviewed their parentage. The Ti-plasmid of the GV strain, pMP90, is a derivative of the pTi-C58 of the C58 isolate [5]. This plasmid carries a gene involved in *trans*-zeatin synthesis close to the *vir*-gene region but outside of the deleted T-DNA region, the *tzs* gene [27]. This suggests that it may be the source of *trans*-zeatin accumulating in infiltrated regions. In order to eliminate the source of bacterial derived *trans*-zeatin, we depleted GVR of its Ti-plasmid (pMP90), and infiltrated the resulting **GV**3101 ‘**c**ured’ strains (abbreviated **GVC**, and labeled with a colony number) into leaf tissue. We then evaluated the ability of these strains to induce the effects observed in GVR. Indeed, the starch staining as well as the green island tests (Figure 3E and 3F) revealed that GVC strains lost the ability to induce pronounced starch accumulation, and no ‘green islands’ were observed during senscence. This suggests a decreased capacity to produce and maintain high cytokinin levels in the treated areas as observed with GVR.Three separately cured GVC lines were analyzed. Microscopic inspection of GVC treated tissue revealed that in all cases stromule frequency was significantly lower than in the positive control infiltrations with GVR (Figure 3A). Infiltrations with the GVR positive control resulted in a stromule frequency of about 35-40%, which is significantly higher than the 7-14% stromules induction following infiltration with GVC strains and the 5% observed in untreated tissue (Figure 3B). All the cured strains induced less than 15% stromules, i.e. a relatively minor increase in stromule frequency relative to non-treated (Figure 3B), which is in the same range as SF values in LBA and LBR treatments (Figure 2E). Stromule length and PNAI results revealed a similar relationship between treatments, in which GVR showed a clear induction of changes, and the three GVC strains were comparable to the non-infiltrated condition (Figure 3C and 3D).

Using starch accumulation and the presence of ‘green islands’ as indicators of cytokinin activity it can be concluded that the loss of the pMP90 and the *tzs* gene in GVC strains leads to a drastic reduction in cytokinin levels. This reduction was accompanied by a drastically reduced impact of GVC on the quantified plastid parameters to a level comparable to LBR treatments. This indicated that the GVR induced effects were indeed caused by a gene product encoded on the pMP90 Ti-plasmid. Additionally, it suggests that the contribution of the GVR genome to the observed reactions can be neglected.

### Addition of tzs to LBA facilitates changes to plastid morphology and increases starch accumulation

**Figure 4:**
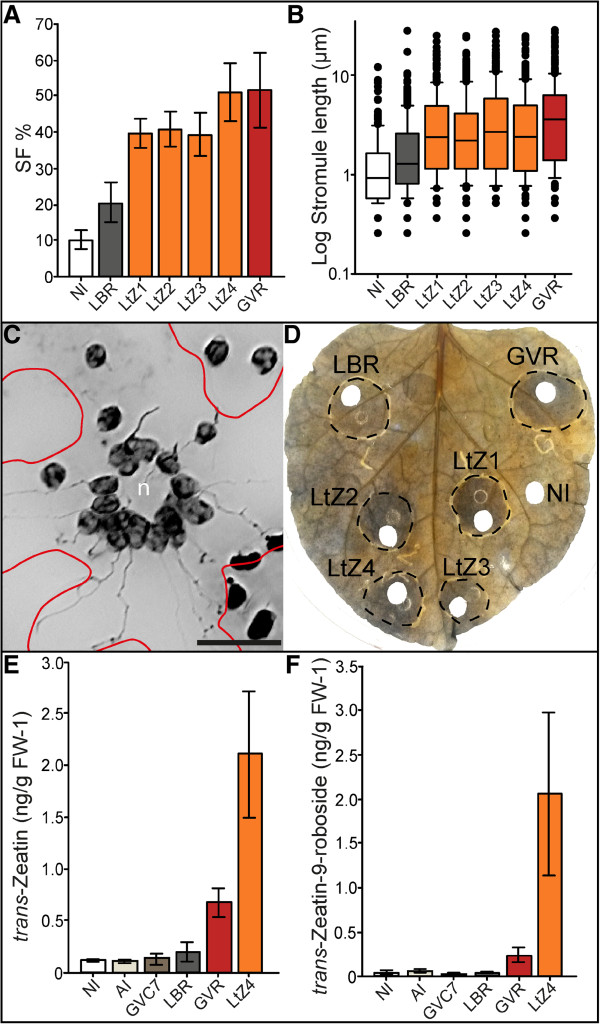
**LBA transformed with 4.3 kb *tzs *gene from pMP90 induces a GVR-like phenotype.** Four different LBA/pLSU-*ptzs*-*tzs* transformants (LtZ1-4) were infiltrated into the lower epidermis of three different *N. benthamiana* plants. **A** Bar graph of average stromule frequency (SF) in non-infiltrated (NI) LBR-infiltrated (LBR), LtZ-infiltrated (LtZ1-4) and GVR-infiltrated (GVR) tissue. Rank sum (NI-LtZ1, 2, 3, 4):U=0, p<0.001; (LtZ1-LBR, LtZ2-LBR): U=7, p<0.001; (LtZ3-LBR): U=8, p<0.001; (LtZ4-LBR): U=4, p<0.001); (LtZ1-GVR): U=39, p=0.061; (LtZ2-GVR): U=40, p=0.069; (LtZ4-GVR): U=62, p=0.829; (LtZ3-GVR): U=32, p=0.039. Sample sizes: n(GV, NI, LtZ1, LtZ2)=12, n(LtZ3, LtZ4)=11. Raw data were arcsine transformed and bars represent back-transformed means. Error bars represent back-transformed 95% confidence intervals. **B** Box plot representing median stromule lengths in non-infiltrated (NI) tissue, LBR-infiltrated (LBR), LtZ-infiltrated (LtZ1-4) and GVR-infiltrated (GVR) tissues. Rank sum (LtZ1-GVR): U=54007, p<0.001; (LtZ2-GVR): U=51988, p<0.001; (LtZ3-GVR): U=53068.5, p=0.015; (LtZ4-GVR): U=50161, p<0.001; (LtZ1-LBR): U=38767, p<0.001; (LtZ2-LBR): U=39391.5, p<0.001; (LtZ3-LBR): U=33777, p<0.001; (LtZ4-LBR): U=35246.5, p<0.001; (LtZ1-NI): U=21007, p<0.001; (LtZ2-NI): U=21005, p<0.001; (LtZ3-NI): U=18181.5, p<0.001; (LtZ4-NI): U=19019.5, p<0.001. Sample sizes: n(NI)=235, n(LBR)=308, n(LtZ1)=360, n(LtZ2)=360, n(LtZ3)=330, n(LtZ4)=330, n(GVR)=360. Boxes contain 50% of data, the median is represented by black line. Error bars represent 90% confidence intervals. **C** ‘Stacked’ fluorescence image of extreme clustering of plastids around the nucleus three days post-infiltration with LtZ4. The cell is outlined in red and the nucleus is indicated by ‘n’. Image was converted to gray scale and inverted to better visualize plastids. Scale=20μm. **D** Iodine stained leaf three days after infiltration with GVR, LtZ strains 1–4, LBR and non-infiltrated control (NI). Infiltrated areas marked by a black dotted outline. **E** LC/MS/MS measurements of absolute *trans*-zeatin in fresh *N. benthamiana* leaf tissue 3 days post-infiltration with AIM buffer (AI), GVC7 (GVC7), LBR (LBR), GVR (GVR), and LtZ4 (LtZ4). Rank sum (GVR-NI, GVR-AI, GVR-GVC7, GVR-LBR, LtZ4-NI, LtZ4-AI, LtZ4-GVC7): U=0, p=0.029; (LtZ4-GVR): U=0, p=0.029. Sample size: n=4 for each. **F** LC/MS/MS measurements of absolute *trans*-zeatin-9-riboside in fresh *N. benthamiana* leaf tissue 3 days post-infiltration with AIM buffer (AI), GVC7 (GVC7), LBR (LBR), GVR (GVR), and LtZ4 (LtZ4). Rank sum (for all pair wise comparisons): U=0, p=0.029). Sample size: n=4 for each.

To specifically test for the involvement of the *tzs* gene (a gene encoded in the pMP90 plasmid) in upregulating cytokinin indicators and plastid-based parameters, we tested if the addition of this gene into LBA would result in a gain of function, GVR/GV-like, phenotype. The *tzs* gene was cloned (coding region plus approximately 2 kb upstream and downstream) from GV and inserted into the small binary vector pLSU [28], simultaneously removing both T-DNA borders and the sequence between to avoid the transfer of the *tzs* from GV to the plant upon infiltration. The vector was then transformed into LBA and four of the subsequent transformants (abbreviated LtZ1-4) were infiltrated into FNR-GFP plants. All four strains induced a drastic increase in stromule frequency relative to NI and LBR strains, with stromule frequencies between 39% and 51% (Figure 4A). Three out of four strains showed no significant difference in SF when compared to GVR control infiltrations (LtZ1, 2 and 4). Median stromule lengths of tissues infiltrated with LtZ1-4 were significantly higher than NI and LBR controls, but significantly lower than GVR (Figure 4B). Although plastid clustering around the nucleus was apparent, and often extreme (see Figure 4C), we were not able to reliably locate the nucleus using DAPI staining or DIC, and so we could not quantify PNAI. Intensity of starch staining of the LtZ-infiltrated regions was comparable to GVR and remained within the infiltrated area as in GVR treatments (Figure 4D).

### Measuring cytokinin accumulation in infiltrated tissues

Up to this point the increase of cytokinin in the plant tissue following infiltration has been indicated by starch staining, and by the maintenance of green islands. To quantify the strain dependant differences in cytokinin production within leaf tissue, liquid chromatography mass spectrometry (LC-MS/MS) was used to directly measure *trans*-zeatin, as well as *trans*-zeatin-9-riboside (Figure 4E and 4F). The same trend was observed for both hormones measured. Cytokinin levels of tissue infiltrated with AIM (infiltration buffer), GVC7, and LBR were below 0.2 ng/g (*trans*-zeatin) and 0.1 ng/g (*trans*-zeatin-9-riboside), while infiltration with both GVR and LtZ showed at least a three-fold increase in cytokinin accumulation (Figure 4E and 4F). Interestingly, LtZ-infiltrated tissues exhibited *trans*-zeatin levels that were about 3 times higher and *trans*-zeatin-9-riboside levels that were about 9 times higher than GVR-infiltrated tissues.

### Tissue infiltrated with cytokinins mimic tissue infiltrated by GV, GVR and LtZ

**Figure 5:**
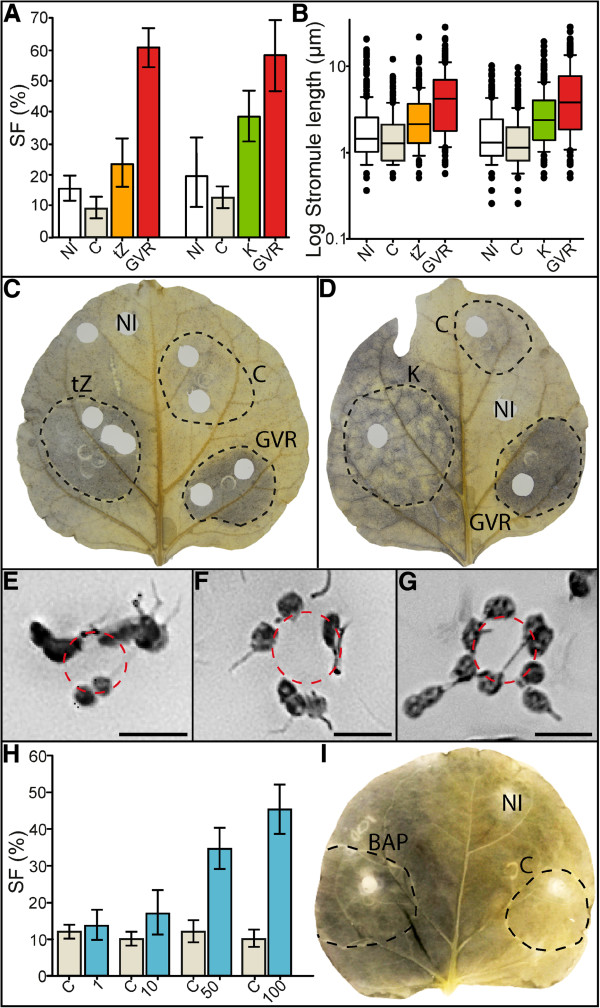
**Treatment with *trans*-zeatin, kinetin or BAP mimics the effects of GVR infiltration. A** Bar graph illustrating stromule frequency (SF) in non-infiltrated (NI), NaOH control-infiltrated (C), *trans*-zeatin-infiltrated (tZ), and kinetin-infiltrated (K) tissues. Rank sum for *trans*-zeatin infiltrated leaf (NI-C): U=13, p=0.017; (NI-GVR, C-GVR, tZ-GVR): U=0, p<0.001; (C-tZ): U=12, p=0.013; (tZ-NI): U = 20, p = 0.077. Rank sum for kinetin infiltrated leaf (NI-K): U=13, p=0.017; (NI-GVR): U=13, p=0.001; (C-K, C-GVR): U=0, p<0.001; (K-GVR): U=11, p=0.01) , (NI-C): U = 27, p = 0.251. Sample size (consecutively): n(NI, C, tZ, NI, C, K, GVR)=9, n(GVR on *trans*-zeatin-infiltrated leaf)=8. **B** Box plot representing median stromule lengths in non-infiltrated (NI), NaOH control-infiltrated (C), *trans*-zeatin-infiltrated (tZ) and kinetin-infiltrated (K) tissues. Rank sum for *trans*-zeatin-infiltrated leaf (NI-tZ): U=24467, p<0.001; (NI-GVR): U=15101.5, p<0.001; (C-GVR): U=9931.5, p<0.001; (C-tZ): U=17141, p<0.001; (tZ-GVR): U=19279, p<0.001; (tZ-NI, tZ-C): U = 23933.5, p = 0.023. . Rank sum for kinetin-infiltrated leaf (NI-K): U=18243, p<0.001; (NI-C): U=24938, p<0.001; (NI-GVR): U=14310.5, p<0.001; (C-GVR): U=11592.5, p<0.001; (C-K): U=14851, p<0.001; (K-GVR); U=23276.5, p<0.001). Sample sizes (consecutively): n(NI)=256, n(C)=213, n(tZ)=249, n(GVR)=240 for the *trans*-zeatin-infiltrated leaf, and n(NI)=239, n(C)=253, n(K)=270, n(GVR)=270 for the kinetin-infiltrated leaf. Boxes contain 50% of data, the median is represented by black line. Error bars represent 90% confidence intervals. **C** Iodine stained leaf showing non-infiltrated (NI) regions as well as regions infiltrated with *trans*-zeatin (tZ), the NaOH control (C), and GVR (GVR). Infiltrated regions outlined with black dotted line. **D** Iodine stained leaf showing non-infiltrated (NI) regions as well as regions infiltrated with kinetin (K), the NaOH control (C), and GVR (GVR). Infiltrated regions outlined with black dotted line. **E-G** Plastids clustered around the nucleus in cells of the lower leaf epidermis following treatments with *trans*-zeatin **(E)**, kinetin **(F)** and BAP **(G)**, respectively. Images have been converted to gray scale and inverted for easier viewing of stromules. Bright field microscopy was used to locate nuclei and position was marked with red-dotted circles. Scale=20 μm. **H** Bar graph illustrating stromule frequency (SF) in NaOH control-infiltrated (C) and BAP-infiltrated (1, 10, 50, 100 μg ml^-1^) tissues. Rank sum (C-1):U=32, p=0.48; (C-10): U=13, p=0.017; (C-50): U=0, p<0.001; (C-100): U=0, p<0.001. n (each treatment)=9. **I** Iodine stained leaf showing non-infiltrated (NI) NaOH control-infiltrated (C), and 100 μg ml^-1^ BAP-infiltrated (BAP) regions. Infiltrated regions outlined with black dotted line. Starch staining spread throughout the leaf, extending beyond the infiltrated area. **A**, **H** Raw data were arcsine transformed and bars represent back-transformed means, and error bars represent back-transformed 95% confidence intervals.

As final proof that cytokinin accumulation and stromule induction are causally linked, even in the absence of all bacterial factors, we infiltrated plants with the hormone to test if we could mimic the effects observed with GVR treatment. For this purpose we infiltrated the tissue with 100 μg ml^-1^ of *trans*-zeatin, administered three times over three consecutive days. 100 μg ml^-1^ kinetin was also tested to determine if stromule induction as well as plastid repositioning is specific to *trans*-zeatin or if this is due to general changes induced by cytokinins. In addition a control solution was also infiltrated to ensure the stress of multiple infiltrations did not induce stromules. After a 3-fold infiltration with each cytokinin and a recovery period of two days we observed, in both instances, an increased stromule frequency (Figure 5A). Average stromule frequency was approximately 23.4% for *trans*-zeatin and 38.6% for kinetin, which were significantly higher than in the NaOH control-infiltrated tissues, but significantly lower than the GVR positive controls, which were found to be close to 60%. Stromule length was also altered by the hormone treatment in a similar way as observed after bacteria treatments, showing a small, but significant increase in stromule length relative to control treatments (Figure 5B). As in tissue infiltrated with GV, GVR and LtZ strains, clustering of plastids around the nucleus was visible in *trans*-zeatin and kinetin treatments (Figure 5E and 5F), but again the efficiency of the DAPI staining was very low so PNAI could not be quantified. Starch also accumulated in a manner reminiscent of GV infiltrations, although staining was not as dark as after the bacterial treatments (Figure 5C and 5D). Although we infiltrated in a restricted region of the leaf (outlined in Figure 5C and 5D with dotted lines) dark starch staining was not limited to the infiltrated region, but spread to adjacent tissue, implying the spread of the hormone. In some cases this affected the non-infiltrated control samples (Figure 5C) providing an explanation for the higher than average values of stromule frequency in the control treatments.

3-fold infiltrations of kinetin and *trans*-zeatin were performed to counteract potential degradation of the cytokinins by the plant, which was suspected after single infiltrations of 100 μg ml^-1^ resulted in inconsistent stromule induction and starch accumulation after 2–3 days (data not shown). To minimize the influence of hormone degradation, single infiltrations were performed using the synthetic cytokinin 6-Benzylaminopurine (BAP). This hormone is broken down much slower than other cytokinins when applied exogenously and can still be detected in plant tissue 6 weeks after infiltration [29]. A gradient of 1 μg ml^-1^, 10 μg ml^-1^, 50 μg ml^-1^ and 100 μg ml^-1^ BAP was infiltrated into tissue and stromule frequency was evaluated. At only 2 days post-infiltration there was a significant induction of stromules at all concentrations except 1 μg ml^-1^, which was comparable to the control infiltration (Figure 5H). 100 μg ml^-1^ BAP-infiltrated tissue had an average SF value of 45%, a SF comparable to that of GV and GVR evaluated at 3 days post-infiltration (compare Figure 5H to Figures 1C, 2E, 3B, 4A). Plastids were also seen to form clusters around nuclei (Figure 5G) and extensive starch staining (Figure 5I), similar to observation made following hormone infiltration and treatment with *tzs* containing bacterial strains. Although the physiological symptoms induced following bacterial infiltrations are not as severe, it appears that direct infiltration with cytokinin is capable of mimicking the effects of *tzs* expression by *A. tumefaciens*.

## Discussion

Stromules are induced under a wide range of biotic and abiotic stresses, and based on our previous observation that the number of stromules increases following infiltration of GVR into *N. benthamiana* leaf tissue [10], we suspected this phenotype may be indicative of cellular changes during *Agrobacterium*-mediated transient assays. Under conditions typical of transient assays (OD_600_ = 0.8, microscopy 2–3 days post-infiltration) we confirmed the previously reported stromule induction by GV3101 (pMP90) (GV) and found that GV triggers alterations to plastid position and starch accumulation. The use of transient assays assumes that the cells observed are displaying authentic sub-cellular behaviour, but these significant changes in chloroplast morphology, position and carbohydrate content indicate that cells assumed to be ‘normal’ might actually be very different from non-infiltrated neighbouring tissue. The determination of the ‘inducing agent’ responsible for such cellular changes was imperative to understanding the magnitude of GV’s impact on plant physiology within transient systems. Based on the observation that infiltrated tissue was displaying symptoms characteristic of increased cytokinins, such as the formation of green islands during senescence and increased starch accumulation, we performed experiments to determine if this was the ‘inducing’ agent responsible for the plastid related changes observed.

### Agrobacterium strain dependant secretion of cytokinins correlates with stromule induction and starch accumulation

Wild type *A. tumefaciens* strains are known to harbour cytokinin synthesis genes, which are transferred with the T-DNA to the plant cell forcing transformed cells to produce cytokinins. In the course of disarming the wildtype *A. tumefaciens* strains for laboratory use, the tumor inducing T-DNA was removed from the wildtype Ti-plasmid, thus removing the ability of the bacteria to induce cytokinin production by the plant. However, some *A. tumefaciens* strains, such as C58 (the wild type ancestor of GV), are capable of secreting a significant amount of cytokinin, specifically *trans*-zeatin, into the culture medium even in the absence of plant cells (concentrations of up to 35 μM measured by [30]). Cytokinin accumulation in culture was found to be the direct result of the activation of the *tzs* (*trans-zeatin synthase*) gene [30], an isopentenyl transferase (IPT) gene situated outside of the wild type T-DNA boundaries, in very close proximity to the *vir* region of the Ti plasmid [30,31]. The *tzs* gene was not removed during the creation of the disarmed Ti-plasmid pMP90 [17] harbored by GV [32]. The ability of GV cells to stay viable in leaf tissue for more than two weeks after infiltration [9], coupled with our observation that infiltrated tissue developed strong physiological symptoms characteristic of high cytokinin levels (Figure 2H and 2K), suggests that GV is also a constant and significant source of cytokinin in plant tissue in the days following infiltration. In support of this hypothesis LC-MS/MS measurements indicated the accumulation of *trans*-zeatin in GV-infiltrated tissues (Figure 4E).

In contrast to GV, we found that LBA (LBA4404), which is an octopine strain, does not induce the same level of starch accumulation (Figure 2K), the green island phenotype (Figure 2H), or any significant increase in either *trans*-zeatin or its riboside (Figure 4E, 4F). This is in line with previously published data suggesting that octopine strains lack the *tzs* gene on their Ti-plasmids, and are thus incapable of secreting high levels of *trans*-zeatin in culture [30,32].

Evidence supporting the role of the *tzs* gene in the production of the ‘inducing agent’ responsible for altering plastid-based parameters came from our curing of GV, which resulted in a drastic decrease in cytokinin indicators and stromule induction. More definitive evidence for the causal link between *tzs* derived cytokinin and stromule induction was derived from the cloning of *tzs* into pLSU and transformation into LBA. The resulting strains (LtZ1-4) increased stromule frequency, stromule length and dark starch staining reminiscent of GV and GVR infiltrations. Additionally, all these symptoms were correlated with an increase in both *trans-*zeatin and its riboside within infiltrated tissues, confirming that the cloned *tzs* gene was functional. The ‘gain-of-function’ phenotype of LBA following addition of a single gene, *tzs*, defines a role for this gene in the production of bacteria-derived cytokinins as well as significant changes to the cell.

After establishing causality between the *tzs* gene and alterations to plastids via the generation of the LtZ strain, causation between cytokinins themselves and the effects observed was established via direct infiltration with three different cytokinins, *trans*-zeatin, kinetin and BAP. Infiltrated cytokinins were found to influence plastid morphology and starch accumulation, but our ability to produce the same magnitude of change as that induced by *tzs* containing *A. tumefaciens* strains was limited. We suggest several possible explanations for this. The first being that it was very difficult to assess what concentration of hormone should be used in our infiltrations, as it was very difficult to determine the amount of cytokinin the bacteria produced to elicit the given response in plant tissue. *A. tumefaciens* anchors to the plant cell wall during infection [33], and secretion of the cytokinin by the immobilized bacteria would likely lead to a highly localized accumulation of cytokinin and a non-homogeneous distribution of hormone within the plant tissue. In addition, free-base cytokinins (like *trans*-zeatin) preferentially accumulate in the chloroplast, leading to the non-uniform distribution of hormone at the cellular level, further hindering the ability to determine their concentration at their point of action [34]. Thus, although our method of measuring whole leaf homogenate provides a good first estimation of the changes in *trans*-zeatin induced by *A.tumefaciens*, it could lead to the underestimation of the absolute amount of cytokinin required to elicit the observed changes to plant physiology. The second problem we faced in attempting to infiltrate hormones directly was that single infiltrations with *trans*-zeatin or kinetin did not consistently induce stromules or starch staining, suggesting their possible inactivation. Cytokinin oxidases, important cytokinin inactivators [35], are actually known to be up-regulated by the presence of the hormone itself [35,36], meaning that upon infiltration with cytokinins we could simultaneously have triggered their increased inactivation. The third issue was the possible diffusion of infiltrated hormones away from the infiltration zone, as indicated by extensive starch staining of infiltrated leaves (Figure 5C, 5D and 5I). This likely resulted in a decrease in the concentration of the hormone in the infiltrated region and additionally prevented us from controlling the amount of hormone in the infiltration zone. Although we could not prevent hormone diffusion, we have used BAP as an alternative to *trans*-zeatin and kinetin in infiltrations, due to an increased resistance to degradation [29]. A gradient of BAP showed that single infiltrations of the hormone with concentrations as low as 10 μg ml^-1^ were capable of significant stromule induction at only two days post-infiltration (Figure 5H). It should be noted that the concentration of *trans*-zeatin measured in the supernatants of C58 cultures by Powell et al. [30] was comparable to this value (35 μM = 7.67 μg ml^-1^). Increasing concentrations of the hormone (1–100 μg ml^-1^) resulted in a corresponding increase in stromule number, thus causally linking the hormone and the formation of these protrusions from the plastid body.

### The expression of the *tzs* gene increases the abundance of multiple cytokinin species

Multiple infiltrations with pure *trans*-zeatin indicated that this hormone is sufficient to mimic the effects of *tzs* expression by the bacteria and induce changes to plastid morphology and position. However, it is important to note that the expression of the *tzs* gene is known to increase the abundance of other cytokinins [37], which could also influence the measured plastid parameters. The *tzs* gene product exhibits catalytic activity specific to both HMBDP (precursor of *trans*-zeatin-type cytokinins) and DMAPP (precursor for iP-type cytokinins) substrates, meaning that this enzyme is capable of acting in multiple cytokinin biosynthesis pathways that produce multiple cytokinin species [37]. The cloning of the C58 *tzs* gene into *E.coli* and subsequent measurements of cytokinins secreted into culture fluids showed that although the increase in *trans*-zeatin was the most obvious (from 11 ngL^-1^ before *tzs* added to 19,900 ngL^-1^ after the addition), there was also a increase in *iso*-pentenyladenine (iP)-type cytokinins (from 120 to 8,700 ngL^-1^), *trans*-ribosylzeatin (from <1 to 236 ngL^-1^) and *iso*-pentenyladenosine (iPA)-type (25 to 85 ngL^-1^) cytokinins [27]. The sensitivity of the cell to the different species seems to depend largely on the ligand preference of the cytokinin receptors present [38,39]. Based on data from *Arabidopsis* and maize it seems that iP and *trans*-zeatin are most often preferred [38,39], but the sensitivity of *N. benthamiana* receptors is unknown. For this reason, although we can say that cytokinins are changing plastid morphology, at this point we cannot speculate further as to which product of the *tzs* gene has the greatest influence on the measured parameters.

### Exposure to *tzs*-derived cytokinins alters cell physiology

Our experiments establish that GV and other *tzs* containing strains are a constant source of significant amounts of cytokinin when infiltrated into leaf tissue, and that this is the cause for the observed changes to plastid morphology in the relatively short time-scale of transient assays. Previous studies have also observed changes to chloroplast structure following exposure to elevated cytokinins. For example, the formation of crystalloids or an ‘amoeboid-like’ morphology is characteristic of plastids in the leaves of stable transgenic tobacco overexpressing cytokinin synthesis genes [34,40]. Although we never observed crystalloids, the description of the ‘amoeboid-like’ plastid structure is reminiscent of our observations of multiple stromules emanating from a single plastid body. Thus, it seems that, although the exposure of plants to *tzs*-derived cytokinins is limited to a few days in the transient assay system, the physiological changes triggered by this short exposure are significant enough to favour the alteration of the plastid membrane in a manner similar to long term exposure.

We have shown that the short duration of GV-based transient assays is also sufficient to induce changes in carbohydrate content, as evident by an increase in starch and soluble sugar (sucrose and glucose) content in infiltrated tissue. This finding is concurrent with literature reports that implicate cytokinins in the regulation of tissue carbohydrate status and carbohydrate partitioning, thereby influencing the source/sink status of a tissue (reviewed in [25,26,41]). This suggests that during a typical transient assay the secretion of *tzs*-derived cytokinins may be significant enough to switch the carbohydrate status tissue from a source to a sink.

The formation of stromules has been frequently observed in tissues considered to be sinks for carbohydrates [42]. This idea is supported by the work of Schattat and Klösgen [43], which showed that treatment with exogenous glucose and sucrose induced stromules in *Arabidopsis thaliana* upper leaf epidermis. This could suggest that the observed stromule induction is a consequence of elevated soluble sugar levels induced by *tzs* cytokinins. However, in contrast to *A. thaliana*, the increases in soluble sugars were not as clearly correlated with an increase in stromule frequency. Despite the absence of the *tzs* gene in LBR, there was a significant increase in soluble sugars that was accompanied by only very small changes to stromule frequency relative to non-infiltrated treatments (Figure 2E, 2I and 2J). Although the increase in sugar level was more pronounced in GVR-infiltrated tissues, a direct connection between elevated sugar and altered plastid morphology in *N. benthamiana* remains to be established.

### Plastid relocation appears independent of stromule formation

The intermediate phenotype of LBR observed during sugar measurements was also observed when PNAI was measured. In contrast to stromule induction, relocation of plastids to the nucleus was not completely abolished in strains lacking the *tzs* gene (LBR or GVC), which resulted in intermediate or GV-like phenotypes (Fig 2G). Plastid proximity to the nucleus and the incidence of stromules has often been discussed as though these two phenomena are functionally related [44-46]. However, clustering of plastids around the nucleus has been reliably observed preceding cell division, and is suggested to ensure unbiased organelle inheritance [47], and this phenomenon may be unrelated to the formation of stromules. It has long been known that treatment with a very minimal amount of exogenous cytokinin (1ug L^-1^) is capable of inducing cell division in tobacco tissue culture [48]. We found that single treatments with *trans*-Zeatin (100 mg ml^-1^) induces plastids to reliably cluster around the nucleus (data not shown), a treatment that did not reliably induce stromules, suggesting that plastid position seems to be independent of stromule formation, and possibly more sensitive to the hormone. A higher sensitivity of PNAI to cytokinins could offer one explanation for the frequent association of plastids with nuclei in some LBA and cured treatments, which occurred in conjunction with low levels of stromule induction.

### Are stromule induction, starch accumulation and plastid relocation pathogen related responses?

Our experiments are not the first to elucidate the effect of disarmed *A. tumefaciens* strains on infiltrated leaf tissue. In two other studies, which focused on plant-pathogen interactions, it was found that infiltration of tobacco with standard disarmed laboratory strains (including GV3101(pMP90)) interferes with subsequent infections with tobacco mosaic virus (TMV) and *Pseudomonas syringae*[8,9]. In addition to reducing infection symptoms, infiltration of ‘disarmed’ *Agrobacterium* strains altered the expression of pathogen responsive gene PR-1 (pathogen related 1), induced chlorosis, inhibited leaf expansion and reduced ABA levels and SA production induced by *P. syringae*[8,9]. Cells cured of the Ti-plasmid elicited these responses to nearly the same extent as the original strains, leading the authors of both studies to the conclusion that these pathogen related responses occur independent of Ti-plasmid encoded gene products [8,9]. In contrast to these Ti-plasmid independent phenotypes, we found stromule formation, plastid relocation and starch accumulation to be highly dependent on Ti-plasmid derived cytokinins. Although we cannot exclude that *Agrobacterium*-derived factors might modulate the induction of these processes to some extent, the fact that we were able to mimic the infiltration of *tzs* harbouring bacteria with hormone solutions shows that other bacteria-derived factors are not essential to induce the observed reactions. Therefore it seems most likely that stromule formation is not a result of a pathogen specific reaction of the cells.

### Evaluating Agrobacterium-mediated transient assays

GV is a favoured laboratory strain, likely due to its high transformation efficiency. In most protocols the bacteria is adjusted to an OD of 0.8, as we did in our experiments, however lower densities have been found to yield sufficient expression of transgenes [49]. This leads to the hypothesis that lowering the concentration of bacteria used during GV-based experiments might reduce the adverse effects of this particular strain, while maintaining a sufficient amount of transient gene expression. Using the induction of stromules as a ‘bio-indicator’ for cytokinin accumulation, we have briefly tested whether there is a concentration of GVR where we observe the transgene, but do not observe stromules. Preliminary results indicate that even at an OD_600_ of 0.01, when very little DsRed2 was detected, GVR can still induce SF values close to 30% 3 days post-infiltration (data not shown). Suggesting that despite a drastic decrease in the amount of bacteria, cytokinin still accumulates to a level sufficient to induce changes to the plastid. Using stromules as an indicator, the viability of this strategy could be further tested for highly expressed and stable transgene products. This may allow for the reduction of the OD to a level where stromules are no longer induced but the visibility of the proteins is maintained.

An alternative strategy to lower the impact of the bacteria-born cytokinins is to limit exposure time by analysing tissue as soon as expression of the transgene is detectable. However studies in *Arabidopsis* have revealed the impact of cytokinin on gene expression and the proteome is widespread and immediate [50-52]. For example, treatment of seedlings with 5 μM exogenous cytokinin results in the differential expression of 82 genes [50] and the differential regulation of 96 proteins within the first 15 minutes [52]. Most of the proteins were localized to the chloroplast (52%), but others were predicted to localize to the cell wall, cytoplasm, ER, nucleus, mitochondria, and vacuole [52]. Although such responses must be evaluated for *N. benthemiana* in the context of transient experiments, it appears that even short-term exposure to low levels of hormone have the potential to affect the composition of multiple cellular compartments, and the influence of bacteria-derived cytokinins during transient assays may be much more extensive than originally hypothesized.

In addition to changing parameters of the infiltration and analysis protocol, an obvious solution is to use an *A. tumefaciens* strain that is devoid of the *tzs* gene. Thus the LBA strain, which lacks the *tzs* gene, may be more appropriate for use in a transient system. However, even this strain induces weak changes to chloroplast morphology, position, and soluble sugar levels. This is most likely due to the accumulation of additional cytokinin species. Both nopaline (e.g. GV and GVR) and octopine *A. tumefaciens* strains (e.g. LBA and LBR) secrete iP, and the production of this cytokinin continues in both strains even after the removal of the Ti-plasmid [31]. Although our data suggests that the largest impact of *A. tumefaciens* is the result of strain-dependant secretion of *tzs*-born cytokinin, other bacterial factors should not be discounted when interpreting the results of transient assays. The variable impact of different bacterial strains observed here highlights the need for appropriate controls that evaluate the impact of each bacterial strain on the process under observation.

## Conclusion

There is no denying that *Agrobacterium*-mediated transient assays provide a valuable tool for the investigation of protein localization and gene expression in the context of living cells. However, whether the cells visualized during these assays represent ‘normal’ cells has been called into question. We have reported that drastic changes to chloroplast shape, position, and carbohydrate status are the result of the strain-dependant secretion of cytokinin into plant tissue by the bacteria. Although we have only examined the effect of transient *A. tumefaciens* infestation on one organelle, altered plastid shape and starch content are indicative of an effect on the physiology of the entire cell. We emphasize the importance of designing appropriate controls to test the influence of the bacteria on the structures under observation, as well as confirming transient assay results via the generation of stable transgenic lines when possible.

## Methods

### Plant material and growth conditions

Plant material was grown on soil under short day conditions (8 h light/16 h dark) at a temperature of approx. 20°C (day and night) and a light intensity of approx. 120 μE m^-2^. Infiltrations were performed in the third or fourth leaf of four to six week old plants grown on soil. For visualization of plastids and stromule morphology transgenic *N. benthamiana* lines expressing a stroma targeted eGFP were used (generation of plants lines described in [10]); chimeric FNR-EGFP described in [15]. To rule out the possibility that observed effects are caused by T-DNA insertion in a certain region of the genome of transgenic plants all experiments were performed in three independent transgenic lines (FNR-EGFP_7-25; FNR-EGFP_6-24; FNR-EGFP_3-20) originating from independent transformation events.

### Bacterial strains and culture

*Agrobacterium* strains used for experiments are listed in Table 1. Both GV [17] and LBA [53], were transformed by electroporation (Bio-Rad, *E.coli* Pulser) with the T-DNA vector pCP60-35S-DsRed2 source described in [10]. This vector harbours a 35S-promotor driven expression cassette expressing untagged DsRed2, and confers resistance to kanamycin in bacteria. *A. tumefaciens* strains were grown on YEB agar plates or YEB liquid medium according to [54] and were supplied with the respective antibiotics (rifampicin 100 μg ml-1; gentamycin 20 μg ml-1; kanamycin 100 μg ml-1; streptinomycin 100 μg ml-1) (Duchefa Biochemie, Haarlem, Netherlands).

### Curing GV3101(pMP90)

In order to cure the *A. tumefaciens* strain GV3101(pMP90) of its Ti-plasmid (pMP90) a modified protocol based on the method described by Engler et al. [55] was used. To cure GVR we grew streaks of single colonies on YEB plates at 37°C for a period of 5 days. The plates contained kanamycin and rifampicin. Gentamycin resistance is specified by the Ti-plasmid, and so was not included in the media, thus eliminating pressure to retain the plasmid (summary of strain antibiotic resistance in Table 1). For selection of cured GV3101 (absence of pMP90) 30 single colonies were picked and transferred to two plates harbouring different antibiotic combinations, one with kanamycin/rifampicin and one with kanamycin/rifampicin/gentamycin, and allowed to grow at room temperature. 12 out of 30 picked colonies showed no growth on gentamycin containing plates after 7 days, indicating potential curing, 3 of these are shown in supplementary Additional file 1: Figure S1A. 4 out of these 12 strains were tested for the presence of the *tzs* gene via PCR (Additional file 1: Figure S1B) with primers specific to the *tzs* gene (Additional file 2: Table S1). Primers specific to the nptIII gene (Additional file 2: Table S1) of the pCP60-35S-DsRed2 acted as a positive PCR control (Additional file 1: Figure S1B), as this vector should have been retained during the curing process (primers from Eurofins MWG Operon, Ebersberg, Germany). As an additional check three of these were chosen for infiltration into *N. benthamiana* and the lower epidermis of screened for expression of DsRed2 using a confocal microscope. Cured lines, presumably lacking the virulence genes contained on the Ti plasmid, were not capable of facilitating DsRed2 expression in plants (Additional file 1: Figure S1E and S1G).

### Cloning tzs from GV and transformation into LBA

A 4.3 kb fragment including the *tzs* coding region and approximately 2 kb upstream and 1.7 kb downstream was amplified using primers specifically designed for RF cloning (described by [56]) of the fragment into the pLSU binary vector (*tzs*4kb_pLSU_F:gctagcgcgcggacaagctaggattggctcaggcagcttcgcagcgaaac, *tzs*4kb_pLSU_R: ttgagacacaacgtggatctaattgctgatagaggagaccagagtaactt). RF cloning was followed by transformation into DH5α and selection on LB containing kanamycin, DNA from four different colonies was isolated and transformed into LBA. Resistant LBA colonies were tested via PCR for the presence of *tzs* using primers internal to the *tzs* coding region (Refer to Additional file 2: Table S1).

### Infiltration of Agrobacteria into N. benthamiana leaves

Infiltrations were carried out according to standard transient expression protocols. 1 ml of the bacterial ‘over night’ culture was pelleted, re-suspended and allowed to incubate in the acetosyringone (Sigma-Aldrich, Deisenhofen, Germany) containing infiltration media (AIM consisting of 10 mM MgCl_2_, 5 mM MES pH 5,3 (both from Roth, Karlruhe, Germany) and 150 μM acetosyringone) prior to infiltration. Following 2 h incubation the optical density of the suspension was adjusted to OD_600nm_ = 0.8. AIM alone (buffer control) and the *A. tumefaciens* strains were infiltrated into the bottom side of the third or fourth leaf of 6–8 week old *N. benthamiana* plants with a needless syringe. To allow for better comparison between the results of independent infiltrations in different leaves, we infiltrated one half of the leaf with the control and the other half with the treatment. After 48–72 hrs leaf discs of the infiltrated areas, as well as a non-infiltrated control were harvested and the lower leaf epidermis was observed using an epifluorescence microscope or confocal microscope.

### Staining and tissue preparation for microscopy

To stain nuclei of *N. benthamiana*, leaf disks were incubated in 20 μM DAPI (Roth, Karlsruhe, Germany) for 10 minutes.

For visualising starch, leaves were excised from plants and placed in petri-dishes containing 100% ethanol (Roth, Karlsruhe, Germany) where they were allowed to de-stain overnight at room temperature. Once fully bleached, leaves were rinsed with water, and 6 mL iodine (Roth, Karlsruhe, Germany) was added to stain starch. After 30 min - 1 hr stained leaves were rinsed and excess stains was removed via suspension of leaves in water for 4-5 hrs at room temperature.

### Measurement of sucrose and glucose concentration

For glucose and sucrose measurements of non-infiltrated, buffer-infiltrated (AIM), as well as bacteria-infiltrated tissue, treatments were administered as described previously. For biological and experimental replicates 3 leaves (3^rd^, 4^th^ and 5^th^) of 7 plants were infiltrated. Each leaf was infiltrated with AIM on one half and the other half went untreated or was infiltrated with one of the bacteria strains. After 48 hours leaf disks were harvested and frozen in liquid nitrogen. Sucrose and glucose quantification was performed as described in [57].

### Cytokinin measurements

Frozen plant material was homogenized to a fine powder with a mixer mill MM301 (Retsch, Haan, Germany) at a frequency of 25 s^-1^ for 50 s with a single 5 mm diameter steel bead in a 2 ml Eppendorf tube. The resulting powder was extracted with 200 μl methanol containing 5 ng each of [^2^H_5_]*trans*-zeatin and [^2^H_5_]*trans*-zeatin 9-riboside (OlChemIm Ltd. Olomouc, Czech Republic) as internal standards. After vigorous shaking for 20 min, the slurry was centrifuged twice at 10,000 g for 5 min. The final supernatant was diluted with 800 μl aqueous formic acid solution (2% v/v) and subjected to solid phase extraction (SPE). The SPE 96-well plate was prepared by distributing dry HR-XC-resin (Macherey-Nagel, Düren, Germany) into the wells of a 96-well filtration plate (50 mg per well). The resin was conditioned by 1 ml of methanol followed by 1 ml of water. In this and all subsequent steps, the liquid was passed through the resin by centrifugation at 250xg for 5 min using a JS5.3 swing-out rotor in an Avanti J-26XP centrifuge (Beckman Coulter, Fullerton, CA, USA). After loading the samples, the resin was washed with 1 ml of water, followed by 1 ml of methanol and eluted with 0.35 M NH_4_OH in methanol into a 96 deep well plate (Roth, Karlsruhe, Germany). The eluates were transferred to 2 ml Eppendorf tubes and the solvent was evaporated under vacuum in Savant SC210A Speed Vac Concentrator at 45°C (ThermoFisher Scientific, Waltham, MA, USA). The dry residue was dissolved in 40 μl 10% (v/v) methanol. After dilution with 40 μl of water and centrifugation at 10,000 g for 10 min, the samples were transferred to autosampler vials for LC-MS/MS analysis.

Separations were performed on a Nucleoshell RP18 column (50 × 3 mm, particle size 2.7 μm; (Macherey-Nagel, Düren, Germany) at 30°C using an Agilent 1290 Infinity HPLC system. Eluents A and B were water and acetonitrile, respectively, each containing 0.2% (v/v) acetic acid. After an initial hold at 2% B for 0.5 min, the percentage of B was increased to 28% over 3 min, further increased to 98% in 0.5 min followed by an isocratic period of 1.5 min at 98% B. The starting conditions were restored within the next 0.5 min, and the column was allowed to re-equilibrate for 1 min at 2% B. The flow rate was set to 0.5 ml/min. The analytes were detected on-line by ESI-MS/MS using an API 3200 triple-quadrupole LC-MS/MS system equipped with an ESI Turbo Ion Spray interface, operated in the positive ion mode (AB Sciex, Darmstadt, Germany). The ion source parameters were set as follows: curtain gas: 50 psi, ion spray voltage: 3500 V, ion source temperature: 650°C, nebulizing and drying gas: 70 psi and 50 psi, respectively. Triple quadrupole scans were acquired in the multiple reaction monitoring mode (MRM) with Q1 and Q3 set at “unit” resolution. Scheduled MRM was performed with a window of 90 s and a target scan time of 0.1 s. Selected MRM transitions and compound specific parameters are given in Supplemental Additional file 3: Table S2.

Peak areas were calculated automatically using the IntelliQuant algorithm of the Analyst 1.6.2 software (AB Sciex, Darmstadt, Germany) and manually adjusted if necessary. All subsequent calculations were performed with Excel (Microsoft Office Professional Plus 2010). Hormones were quantified using the internal standards.

### Hormone treatments

Stock solutions of *trans*-zeatin and kinetin (both from Duchefa Biochemie, Haarlem, Niederlande) were made by first dissolving powdered hormone in 0.5 mL 1 N NaOH (Roth, Karlsruhe, Germany). These stock solutions were then used to prepare the 100 μg ml^-1^ working solutions that were used for subsequent infiltrations. A control infiltration was performed that contained NaOH solution at the same concentration (2.28 mM NaOH) as in the treatment condition (basic solution used to increase solubility of hormones). *trans*-zeatin and kinetin were administered with a needless syringe via the same method outlined for *A. tumefaciens* infiltration. Solutions were infiltrated 3 times into the same area of the leaf over the course of 3 consecutive days, and microscopy was performed 2 days after the final infiltration. The procedure was the same for BAP gradient infiltrations (1, 10, 50 and 100 mg L^-1^), except that infiltrations were performed only once, with microscopy completed 2 days post-infiltration. Infiltrated control solutions contained NaOH in same concentration as in treatment condition.

### Microscopy and image processing

Confocal microscopy was performed with an Axiovert mot LSM 510 from Carl Zeiss GmbH, Jena, Germany. DsRed2 and chlorophyll were imaged simultaneously in separate channels. DsRed2 was excited by a 543 nm laser line and the emitted light was filtered by a 560 – 615 nm band pass filter. Chlorophyll fluorescence was excited by a 633 nm laser line and the emitted fluorescence was filtered by an 650 nm long pass filter.

Epifluorescence images were taken with a non-motorised Axioskop2 Zeiss GmbH, Jena, Germany equipped with an Axiocam HRc CCD-Camera (Carl Zeiss, Jena, Germany). Fluorescence illumination was realised with an HBO100 Lamp house equipped with a mercury light bulb (HBO 100 W/2 from Osram, Munich, Germany). EGFP, DsRed2 and DAPI were imaged using the following filter from AHF-Analysetechnik (Tübingen, Germany): DsRed/GFP (F51-019), DsRed (F46-005), endowGFP (F41-017), DAPI/GFP (F51-012).

### Image processing and image analysis

Epifluorescence images were taken as z-stacks. The series of images was exported as a set of individual ‘.tif’ files. The exported images were sharpened in Adobe Photoshop CS6 by applying an unsharpen mask to the single images. Images of one z-stack were subsequently flattened to obtain single images with an extended depth of focus. For the stacking process the free software package CombineZPBatch [58] was utilized, as outlined in [59]. For presentation purposes Epifluorescence images were converted to black and white, and inverted, allowing for better viewing of stromules on printed media. Therefore dual-band filter set images lose their colour difference of DAPI stain or DsRed2 to eGFP. However the nucleus can still easily be identified in the images due to its size and position.

Stromule number, stromule lengths and the association of plastids with the nucleus were counted using the ‘cell counter’ plugin and the ‘measure’ feature of Fiji [60]. Counts and length measurements were made per image neglecting plastids in guard cells. For stromule frequency every fully imaged epidermal plastid was counted. The mean stromule frequency for each treatment represents an average of counts from 9 different images (n = 9), each containing approximately 250–350 plastids (each treatment is represented by 2000 to 3000 plastids). For stromule length measurements 30 stromules were measured per image. In order to avoid biased measurements, stromules were measured in a rectangle in the upper left corner of the respective images. We considered the formation of short stromule branches as being independent from the elongation of the main stromule body. Based on this assumption stromule measurements were made on the longest stromule branches.

### Statistical analysis

For each treatment three different plants from three independent FNR-EGFP lines (3, 6, and 7) were infiltrated, and one disk was punched from the infiltrated region. 3 images were taken per leaf disk, for a total of 9 images. Due to similarity between data sets from the different plant lines the data of the repeats were pooled. Stromule frequency (SF%) represents the proportion of plastids with one or more stromules. For calculation of SF% the number of plastids with stromules was counted and divided by the total number of plastids. The resulting data was arcsin transformed, and statistical analysis was performed on the transformed data. 95% confidence intervals and arithmetic averages were calculated, and back-transformed data was represented in bar graphs (transformations completed using Excel). We introduced PNAI to describe the absolute number of plastids in close association with a given nucleus. Stromule length and PNAI were represented as medians and box plots to better show the distribution of the data.

Glucose and sucrose concentration were obtained from 7 leaves (n = 7) per treatment. In each case the AIM control was infiltrated into the same leaf as one of the treatments, AIM and treatment conditions of each of the 7 leaves was pooled and averaged (arithmetic average). To describe variance the standard deviation was calculated and T-tests were performed to compare normally distributed data, while the Mann–Whitney Rank Sum Test was used to compare data that failed a Normality Test, or Equal Variance Test. Comparisons were made between AIM and treatment conditions administered to the same set of leaves. Sigma Plot was used for statistical analysis of all data.

## Competing interests

The authors declare that they have no competing interests.

## Authors’ contributions

JLE and MHS were responsible for experimental design, analysis, and wrote the article; JZ performed hormone measurements under the supervision of SA; DG performed sugar measurements under the supervision of SJR; JM and RBK contributed advice on the manuscript and technical support.

## Supplementary Material

Additional file 1: Figure S1 Evidence of successful curing of GVR of (pMP90). Click here for file

Additional file 2: Table S1 Primers for amplification of *tzs* and *nptII*. Click here for file

Additional file 3: Table S2 MS parameters for MRM-transitions. Click here for file

## References

[B1] McCullen CA Binns AN Agrobacterium tumefaciens and plant cell interactions and activities required for interkingdom macromolecular transfer Annu Rev Cell Dev Biol 2006 22 101 127 10.1146/annurev.cellbio.22.011105.102022 16709150

[B2] Escobar MA Dandekar AM Agrobacterium tumefaciens as an agent of disease Trends Plant Sci 2003 8 380 386 10.1016/S1360-1385(03)00162-6 12927971

[B3] Gelvin SB Agrobacterium-mediated plant transformation: the biology behind the “gene-jockeying” tool Microbiol Mol Biol Rev 2003 67 16 37 10.1128/MMBR.67.1.16-37.2003 12626681 PMC150518

[B4] Zhu J Oger PM Schrammeijer B Hooykaas PJJ Farrand SK Winans SC The bases of crown gall tumorigenesis J Bacteriol 2000 182 3885 3895 10.1128/JB.182.14.3885-3895.2000 10869063 PMC94570

[B5] Hellens RP Edwards EA Leyland NR Bean S Mullineaux PM pGreen: a versatile and flexible binary Ti vector for Agrobacterium-mediated plant transformation Plant Mol Biol 2000 42 819 832 10.1023/A:1006496308160 10890530

[B6] Lee L-Y Gelvin SB T-DNA binary vectors and systems Plant Physiol 2008 146 325 332 18250230 10.1104/pp.107.113001 PMC2245830

[B7] Kuta DD Tripathi L Agrobacterium-induced hypersensitive necrotic reaction in plant cells: a resistance response against Agrobacterium-mediated DNA transfer Afr J Biotechnol 2005 4 752 757

[B8] Rico A Bennett MH Forcat S Huang WE Preston GM Agroinfiltration reduces ABA levels and suppresses Pseudomonas syringae-elicited salicylic acid production in Nicotiana tabacum PLoS One 2010 5 e8977 10.1371/journal.pone.0008977 20126459 PMC2813289

[B9] Pruss GJ Nester EW Vance V Infiltration with Agrobacterium tumefaciens induces host defense and development-dependent responses in the infiltrated zone MPMI 2008 21 1528 1538 10.1094/MPMI-21-12-1528 18986249

[B10] Schattat MH Griffiths S Mathur N Barton K Wozny MR Dunn N Greenwood JS Mathur J Differential coloring reveals that plastids do not form networks for exchanging macromolecules Plant Cell 2012 24 1465 1477 10.1105/tpc.111.095398 22474180 PMC3398557

[B11] Hanson MR Sattarzadeh A Dynamic morphology of plastids and stromules in angiosperm plants Plant Cell Environ 2008 31 646 657 10.1111/j.1365-3040.2007.01768.x 18088332

[B12] Gunning BES Plastid stromules: video microscopy of their outgrowth, retraction, tensioning, anchoring, branching, bridging, and tip-shedding Protoplasma 2005 225 33 42 10.1007/s00709-004-0073-3 15868211

[B13] Kwok EY Hanson MR Stromules and the dynamic nature of plastid morphology J Microsc 2004 214 124 137 10.1111/j.0022-2720.2004.01317.x 15102061

[B14] Mathur J Mammone A Barton KA Organelle extensions in plant cells J Integr Plant Biol 2012 54 851 867 23046073 10.1111/j.1744-7909.2012.01175.x

[B15] Marques JP Schattat MH Hause G Dudeck I Klösgen RB In vivo transport of folded EGFP by the DeltapH/TAT-dependent pathway in chloroplasts of Arabidopsis thaliana J Exp Bot 2004 55 1697 1706 10.1093/jxb/erh191 15208333

[B16] Schattat M Barton K Mathur J Correlated behavior implicates stromules in increasing the interactive surface between plastids and ER tubules Plant Signal Behav 2011 6 715 718 10.4161/psb.6.5.15085 21448009 PMC3172846

[B17] Koncz C Schell J The promoter of TL-DNA gene 5 controls the tissue-specific expression of chimaeric genes carried by a novel type of Agrobacterium binary vector Mol Gen Genet 1986 204 383 396 10.1007/BF00331014

[B18] Schattat M Barton K Baudisch B Klösgen RB Mathur J Plastid stromule branching coincides with contiguous endoplasmic reticulum dynamics Plant Physiol 2011 155 1667 1677 10.1104/pp.110.170480 21273446 PMC3091094

[B19] Sparkes IA Teanby NA Hawes C Truncated myosin XI tail fusions inhibit peroxisome, Golgi, and mitochondrial movement in tobacco leaf epidermal cells: a genetic tool for the next generation J Exp Bot 2008 59 2499 2512 10.1093/jxb/ern114 18503043 PMC2423659

[B20] Ketelaar T Anthony RG Hussey PJ Green fluorescent protein-mTalin causes defects in actin organization and cell expansion in arabidopsis and inhibits actin depolymerizing factor’s actin depolymerizing activity in vitro Plant Physiol 2004 136 3990 3998 10.1104/pp.104.050799 15563618 PMC535831

[B21] Holweg CL Living markers for actin block myosin-dependent motility of plant organelles and auxin Cell Motil Cytoskeleton 2007 64 69 81 10.1002/cm.20164 17009330

[B22] Wingler A Roitsch T Metabolic regulation of leaf senescence: interactions of sugar signalling with biotic and abiotic stress responses Plant Biol 2008 10 50 62 18721311 10.1111/j.1438-8677.2008.00086.x

[B23] Miyazawa Y Sakai A Miyagishima S Takano H Kawano S Kuroiwa T Auxin and cytokinin have opposite effects on amyloplast development and the expression of starch synthesis genes in cultured bright yellow-2 tobacco cells Plant Physiol 1999 121 461 469 10.1104/pp.121.2.461 10517837 PMC59408

[B24] Enami K Ozawa T Motohashi N Nakamura M Tanaka K Hanaoka M Plastid-to-nucleus retrograde signals are essential for the expression of nuclear starch biosynthesis genes during amyloplast differentiation in tobacco BY-2 cultured cells Plant Physiol 2011 157 518 530 10.1104/pp.111.178897 21771917 PMC3165897

[B25] Roitsch T Ehness R Regulation of source/sink relations by cytokinins Plant Growth Regul 2000 32 359 367 10.1023/A:1010781500705

[B26] Werner T Holst K Pors Y Guivarc’h A Mustroph A Chriqui D Grimm B Schmulling T Cytokinin deficiency causes distinct changes of sink and source parameters in tobacco shoots and roots J Exp Bot 2008 59 2659 2672 10.1093/jxb/ern134 18515826 PMC2486470

[B27] Beaty JS Powell GK Lica L Regier DA Macdonald EMS Hommes NG Morris RO Tzs, a nopaline Ti plasmid gene from Agrobacterium tumefaciens associated with trans-zeatin biosynthesis Mol Gen Genet 1986 203 274 280 10.1007/BF00333966

[B28] Lee S Su G Lasserre E Aghazadeh MA Murai N Small high-yielding binary Ti vectors pLSU with co-directional replicons for Agrobacterium tumefaciens-mediated transformation of higher plants Plant Sci 2012 187 49 58 22404832 10.1016/j.plantsci.2012.01.012

[B29] Werbrouck S Strnad M VanOnckelen HA Debergh PC Meta-topolin, an alternative to benzyladenine in tissue culture? Physiol Plant 1996 98 291 297

[B30] Powell GK Hommes NG Kuo J Castle LA Morris RO Inducible expression of cytokinin biosynthesis in Agrobacterium tumefaciens by plant phenolics MPMI 1988 1 235 242 10.1094/MPMI-1-235 2980282

[B31] Regier DA Morris RO Secretion of trans-zeatin by Agrobacterium tumefaciens: a function determined by the nopaline Ti plasmid Biochem Biophys Res Commun 1982 104 1560 1566 10.1016/0006-291X(82)91429-2 7073755

[B32] Han Z-F Hunter DM Sibbald S Zhang J-S Tian L Biological activity of the tzs gene of nopaline Agrobacterium tumefaciens GV3101 in plant regeneration and genetic transformation MPMI 2013 26 1359 1365 10.1094/MPMI-04-13-0106-R 24088018

[B33] Matthysse AG Holmes KV Gurlitz RH Elaboration of cellulose fibrils by Agrobacterium tumefaciens during attachment to carrot cells J Bacteriol 1981 145 583 595 7462151 10.1128/jb.145.1.583-595.1981 PMC217308

[B34] Polanská L Vicánková A Nováková M Malbeck J Dobrev PI Brzobohaty B Vankova R Machácková I Altered cytokinin metabolism affects cytokinin, auxin, and abscisic acid contents in leaves and chloroplasts, and chloroplast ultrastructure in transgenic tobacco J Exp Bot 2007 58 637 649 17175552 10.1093/jxb/erl235

[B35] Brugière N Jiao S Hantke S Zinselmeier C Roessler JA Niu X Jones RJ Habben JE Cytokinin oxidase gene expression in maize is localized to the vasculature, and is induced by cytokinins, abscisic acid, and abiotic stress Plant Physiol 2003 132 1228 1240 10.1104/pp.102.017707 12857805 PMC167063

[B36] Motyka V Vankova R Capkova V Petrasek J Kaminek M Schmülling T Cytokinin-induced upregulation of cytokinin oxidase activity in tobacco includes changes in enzyme glycosylation and secretion Physiol Plant 2003 117 11 21 10.1034/j.1399-3054.2003.1170102.x

[B37] Ueda N Kojima M Suzuki K Sakakibara H Agrobacterium tumefaciens tumor morphology root plastid localization and preferential usage of hydroxylated prenyl donor is important for efficient gall formation Plant Physiol 2012 159 1064 1072 10.1104/pp.112.198572 22589470 PMC3387694

[B38] Yonekura-Sakakibara K Kojima M Yamaya T Sakakibara H Molecular characterization of cytokinin-responsive histidine kinases in maize. Differential ligand preferences and response to cis-zeatin Plant Physiol 2004 134 1654 1661 10.1104/pp.103.037176 15064375 PMC419839

[B39] Stolz A Riefler M Lomin SN Achazi K Romanov GA Schmülling T The specificity of cytokinin signalling in Arabidopsis thaliana is mediated by differing ligand affinities and expression profiles of the receptors Plant J 2011 67 157 168 10.1111/j.1365-313X.2011.04584.x 21426428

[B40] Synková H Schnablová R Polanská L Husák M Siffel P Vácha F Malbeck J Machácková I Nebesárová J Three-dimensional reconstruction of anomalous chloroplasts in transgenic ipt tobacco Planta 2006 223 659 671 10.1007/s00425-005-0119-6 16160843

[B41] Hwang I Sheen J Müller B Cytokinin signaling networks Annu Rev Plant Biol 2012 63 353 380 10.1146/annurev-arplant-042811-105503 22554243

[B42] Waters MT Fray RG Pyke KA Stromule formation is dependent upon plastid size, plastid differentiation status and the density of plastids within the cell Plant J 2004 39 655 667 10.1111/j.1365-313X.2004.02164.x 15272881

[B43] Schattat MH Klösgen RB Induction of stromule formation by extracellular sucrose and glucose in epidermal leaf tissue of Arabidopsis thaliana BMC Plant Biol 2011 11 115 10.1186/1471-2229-11-115 21846357 PMC3167769

[B44] Kwok EY Hanson MR Plastids and stromules interact with the nucleus and cell membrane in vascular plants Plant Cell Rep 2004 23 188 195 10.1007/s00299-004-0824-9 15252692

[B45] Caplan JL Mamillapalli P Burch-Smith TM Czymmek K Dinesh-Kumar SP Chloroplastic protein NRIP1 mediates innate immune receptor recognition of a viral effector Cell 2008 132 449 462 10.1016/j.cell.2007.12.031 18267075 PMC2267721

[B46] Krenz B Jeske H Kleinow T The induction of stromule formation by a plant DNA-virus in epidermal leaf tissues suggests a novel intra- and intercellular macromolecular trafficking route Front Plant Sci 2012 3 291 23293643 10.3389/fpls.2012.00291 PMC3530832

[B47] Sheahan MB Rose RJ McCurdy DW Organelle inheritance in plant cell division: the actin cytoskeleton is required for unbiased inheritance of chloroplasts, mitochondria and endoplasmic reticulum in dividing protoplasts Plant J 2003 37 379 390 14731258 10.1046/j.1365-313x.2003.01967.x

[B48] Miller CO Skoog F Von Saltza MH Strong FM Kinetin, a cell division factor from deoxyribonucleic acid J Am Chem Soc 1955 77 1392 1392 10.1021/ja01610a105

[B49] Sparkes IA Runions J Kearns A Hawes C Rapid, transient expression of fluorescent fusion proteins in tobacco plants and generation of stably transformed plants Nat Protoc 2006 1 2019 2025 10.1038/nprot.2006.286 17487191

[B50] Brenner WG Romanov GA Köllmer I Bürkle L Schmülling T Immediate-early and delayed cytokinin response genes of Arabidopsis thaliana identified by genome-wide expression profiling reveal novel cytokinin-sensitive processes and suggest cytokinin action through transcriptional cascades Plant J 2005 44 314 333 10.1111/j.1365-313X.2005.02530.x 16212609

[B51] Brenner WG Ramireddy E Heyl A Schmuelling T Gene regulation by cytokinin in Arabidopsis Front Plant Sci 2012 3 8 22639635 10.3389/fpls.2012.00008 PMC3355611

[B52] Cerny M Dycka F Bobalova J Brzobohaty B Early cytokinin response proteins and phosphoproteins of Arabidopsis thaliana identified by proteome and phosphoproteome profiling J Exp Bot 2011 62 921 937 10.1093/jxb/erq322 20974740 PMC3022391

[B53] Hoekema A Hirsch PR Hooykaas PJJ Schilperoort RA A binary plant vector strategy based on separation of vir-region and T-region of the Agrobacterium-tumefaciens Ti-plasmid Nature 1983 303 179 180 10.1038/303179a0

[B54] Wise AA Liu Z Binns AN Wang K Culture and Maintenance of Agrobacterium Strains Agrobacterium Protocols, Volume 1 2006 2 Totowa: Humana Press 2 13

[B55] Engler G Holsters M Van Montagu M Schell J Hernalsteens JP Schilperoort Agrocin 84 sensitivity: a plasmid determined property in Agrobacterium tumefaciens Mol Gen Genomics 1975 138 345 349 10.1007/BF00264804 1152843

[B56] van den Ent F Löwe J RF cloning: a restriction-free method for inserting target genes into plasmids J Biochem Biophys Methods 2006 67 67 74 10.1016/j.jbbm.2005.12.008 16480772

[B57] Coneva V Guevara D Rothstein SJ Colasanti J Transcript and metabolite signature of maize source leaves suggests a link between transitory starch to sucrose balance and the autonomous floral transition J Exp Bot 2012 63 5079 5092 10.1093/jxb/ers158 22791826 PMC3430989

[B58] Hadley A My Software to Combine Pictures to Increase Depth of Field 2006 http://www.hadleyweb.pwp.blueyonder.co.uk/

[B59] Schattat MH Klösgen RB Improvement of plant cell microscope images by use of “depth of field” - extending software Endocytobiosis Cell Res 2009 19 11 19

[B60] Schindelin J Arganda-Carreras I Frise E Kaynig V Longair M Pietzsch T Preibisch S Rueden C Saalfeld S Schmid B Tinevez J-Y White DJ Hartenstein V Eliceiri K Tomancak P Cardona A Fiji: an open-source platform for biological-image analysis Nat Methods 2012 9 676 682 10.1038/nmeth.2019 22743772 PMC3855844

