# Supplementary material for: Agrobacterium-derived cytokinin influences plastid morphology and starch accumulation in Nicotiana benthamiana during transient assays
Source: BMC Plant Biol. 2014 May 9;14:127. doi: 10.1186/1471-2229-14-127 (PMC4062310; doi:10.1186/1471-2229-14-127)
Supplement: Additional file 1: Figure S1 — Evidence of successful curing of GVR of (pMP90). [file 1471-2229-14-127-S1.pdf]

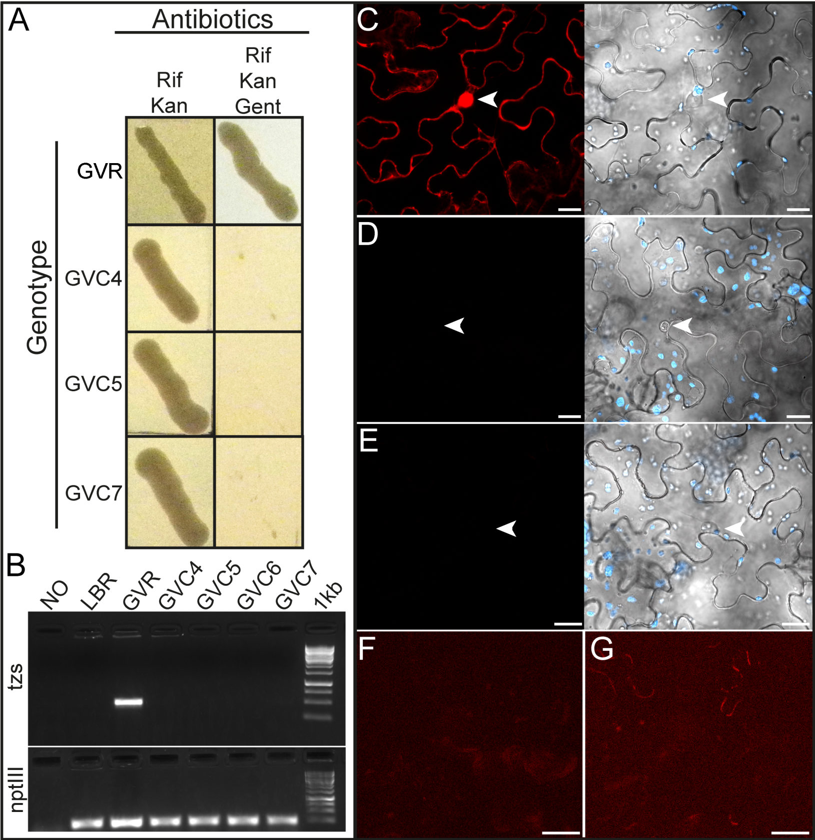
A
Antibiotics
Rif
Kan
Rif
Kan
Gent
Genotype
GVR
GVC4
GVC5
GVC7
B
NO
LBR
GVR
GVC4
GVC5
GVC6
GVC7
1kb
tzs
nptIII
C
D
E
F
G

Supplemental Figure 1. Evidence of successful curing of GVR of (pMP90)

A *A. tumefaciens* plated on YEB with antibiotics (Rif=rifampicin, Kan=kanamycin, Gent=gentamycin) to show the loss of gentamycin resistance in putative cured strains GVC4, 5 and 7. GVR was plated as a control.

B The top panel is a colony PCR confirming the loss of the *tzs* gene from 4 *A. tumefaciens* strains (GVC4-7) following curing. Negative controls included a no DNA control (NO) and LBR, while GVR served as the positive control. As a PCR control the same set of colonies was used in a second PCR reaction (lower panel) where primers specific to the *nptIII* gene present in the pCP60-35S-dsRed2 were used (this vector should be present in all strains used and so should produce a product in all cases except the no DNA control).

C-E Confocal images of *N. benthamiana* lower epidermis. Left panel is the DsRed channel, and right panel is the multi-channel image (bright field and chlorophyll, which is falsely colored blue) of the same cell. The position of the nuclei is indicated by white arrows.

C GVR-infiltrated positive control accumulating DsRed2 in the nucleus (indicated by a white arrow) and the cytoplasm (outlines cells in red).

D Non-infiltrated negative control showing no expression of DsRed2.

E GVC4-infiltrated tissue showing no expression of DsRed2.

F Non-infiltrated tissue, DsRed2 channel under high gain.

G GVC4-infiltrated tissue, DsRed channel under high gain.
