# Supplementary material for: Agrobacterium-derived cytokinin influences plastid morphology and starch accumulation in Nicotiana benthamiana during transient assays
Source: BMC Plant Biol. 2014 May 9;14:127. doi: 10.1186/1471-2229-14-127 (PMC4062310; doi:10.1186/1471-2229-14-127)
Supplement: Additional file 2: Table S1 — Primers for amplification of tzs and nptII. [file 1471-2229-14-127-S2.doc]

**Additional file 1: Table S1. Primers for colony PCR confirming the loss of the *tzs* gene from putative GVC strains.**

| Primer | Sequence (5’-3’) |
| --- | --- |
| tzs_internal_F | gatccaaatcgcacaagaaa |
| tzs_internal_R | tccgtcgatatcttccaaa |
| nptIII_internal_F | aaaaactgatcgaaaaataccg |
| nptIII_internal_R | ggcctcactcatgagcagatt |

List of primers used for colony PCR (column 1), including those used to test for the presence of the gene (rows 1 and 2), and those used for the PCR positive control (rows 3 and 4). Sequences of primers displayed in column 2. Primers were designed using Primer 3.
