# Supplementary material for: Agrobacterium-derived cytokinin influences plastid morphology and starch accumulation in Nicotiana benthamiana during transient assays
Source: BMC Plant Biol. 2014 May 9;14:127. doi: 10.1186/1471-2229-14-127 (PMC4062310; doi:10.1186/1471-2229-14-127)
Supplement: Additional file 3: Table S2 — MS parameters for MRM-transitions. [file 1471-2229-14-127-S3.doc]

**Additional file 2: Table S2. MS parameters for MRM-transitions**

| Hormone | MRM transitions | Declustering potential (DP), V | Entrance potential (EP), V | Cell entrance potential (CEP), V | Collision potential (CE), V | Cell exit potential (CEX), V |
| --- | --- | --- | --- | --- | --- | --- |
| *trans*-zeatin | **220→136** | 41 | 3.5 | 28 | 23 | 4 |
|  | *220→119* | 41 | 3.5 | 28 | 43 | 4 |
| [^2^H_5_]*trans*-zeatin | **225→136** | 41 | 3.5 | 28 | 23 | 4 |
|  |  |  |  |  |  |  |
| *trans*-zeatin 9-riboside | **352→220** | 61 | 10.5 | 28 | 23 | 4 |
|  | *352→136* | 61 | 10.5 | 28 | 43 | 4 |
| [^2^H_5_]*trans*-zeatin 9-riboside | **357→224** | 61 | 10.5 | 28 | 23 | 4 |
|  |  |  |  |  |  |  |
|  |  |  |  |  |  |  |

Quantifier and qualifier transitions are indicated in bold and italics, respectively
